# AGGF1 therapy inhibits thoracic aortic aneurysms by enhancing integrin α7-mediated inhibition of TGF-β1 maturation and ERK1/2 signaling

**DOI:** 10.1038/s41467-023-37809-x

**Published:** 2023-04-20

**Authors:** Xingwen Da, Ziyan Li, Xiaofan Huang, Zuhan He, Yubing Yu, Tongtong Tian, Chengqi Xu, Yufeng Yao, Qing K. Wang

**Affiliations:** 1 Center for Human Genome Research, Key Laboratory of Molecular Biophysics of the Ministry of Education, College of Life Science and Technology, Huazhong University of Science and Technology, Wuhan, P. R. China; 2 Department of Cardiovascular Surgery, Union Hospital, Tongji Medical College, Huazhong University of Science and Technology, Wuhan, P. R. China; 3 Institute of Medical Genomics and School of Biomedical Sciences, Shandong First Medical University and Shandong Academy of Medical Sciences, Jinan, P. R. China

**Keywords:** Growth factor signalling, Aneurysm, Chronic inflammation

## Abstract

Thoracic aortic aneurysm (TAA) is a localized or diffuse dilatation of the thoracic aortas, and causes many sudden deaths each year worldwide. However, there is no effective pharmacologic therapy. Here, we show that AGGF1 effectively blocks TAA-associated arterial inflammation and remodeling in three different mouse models (mice with transverse aortic constriction, *Fbn1*^*C1041G/+*^ mice, and β-aminopropionitrile-treated mice). AGGF1 expression is reduced in the ascending aortas from the three models and human TAA patients. *Aggf1*^*+/-*^ mice and vascular smooth muscle cell (VSMC)-specific *Aggf1*^*smcKO*^ knockout mice show aggravated TAA phenotypes. Mechanistically, AGGF1 enhances the interaction between its receptor integrin α7 and latency-associated peptide (LAP)-TGF-β1, blocks the cleavage of LAP-TGF-β1 to form mature TGF-β1, and inhibits Smad2/3 and ERK1/2 phosphorylation in VSMCs. Pirfenidone, a treatment agent for idiopathic pulmonary fibrosis, inhibits TAA-associated vascular inflammation and remodeling in wild type mice, but not in *Aggf1*^*+/-*^ mice. In conclusion, we identify an innovative AGGF1 protein therapeutic strategy to block TAA-associated vascular inflammation and remodeling, and show that efficacy of TGF-β inhibition therapies require AGGF1.

## Abstract (web summary)

Thoracic aortic aneurysm (TAA) causes many sudden deaths each year, however, no effective drug treatment is available. Here, the authors show that AGGF1 protein therapy attenuates TAA in three different mouse models through integrin α7-mediated inhibition of TGF-β1 maturation and ERK1/2 signalling.

## Introduction

Aortic aneurysm causes about 200,000 deaths per year worldwide^1,2^. An aneurysm occurs due to the dilatation of blood vessels at different locations. The mortality is as high as 90% with massive hemorrhages caused by aneurysm rupture, and more than 50% even without rupture^3,4^. Thoracic aortic aneurysm (TAA) is a localized or diffuse dilatation of the thoracic aortas, and can occur from the root of the aortas to the descending thoracic aortas^5^. TAA is a potentially fatal disease that causes a substantial number of sudden deaths each year worldwide, and its incidence is increasing as from 2002 to 2014, its prevalence increased from 3.5 per 100,000 to 7.6 per 100,000^6^. Aneurysms including TAA are diagnosed with imaging technologies, and the main treatment strategies include surgical removal and vascular reconstruction. However, the surgical procedures have adverse effects, and may cause aneurysm rupture, and primary or secondary infection^7^. To date, no effective pharmacologic treatment option exists for TAA.

High blood pressure is a major risk factor for the development of a variety of aneurysms, such as thoracic aortic aneurysms (TAA), aortic dissection, abdominal aortic aneurysms, and cerebral aneurysms^8,9^. The underlying molecular mechanism is poorly understood, however, vascular inflammation and remodeling play a fundamentally important role in the pathogenesis of aortic aneurysms. Extensive vascular remodeling occurs in an ascending aortic aneurysm, which is a life-threatening disease due to the risk of aortic rupture^10^. Kuang et al. showed that transverse aortic constriction (TAC) induced the formation of aneurysms in the ascending aortas and right carotid artery independent of the structure or function of the blood vessels, resulting in the development of a mouse model for aneurysms similar to the commonly used angiotensin II (ANG II)-induced model^11^. Vascular inflammation and remodeling are an adaptive response to various physiological and pathological stimuli, including aging, high blood pressure, mechanical forces, inflammatory cytokines, and hormones^12^. On the other hand, in addition to aortic aneurysms, vascular remodeling is also involved in the pathogenesis of a variety of other diseases, including hypertension, atherosclerosis, nephrosclerosis, stroke, and other cardiovascular, cerebrovascular, and metabolic disorders^12,13^. Despite the common association between vascular remodeling and aortic aneurysms, the mechanisms triggering the remodeling of arteries have not been fully elucidated, and TAC may provide a model for exploring such mechanisms. Moreover, there are no effective drug-based therapies that can block vascular inflammation and remodeling, which highlights an urgent unmet need for clinical practice.

In addition to TAC models, there are two other animal models for TAA. The first model is the knockin (KI) mice with a pathogenic variant p.C1041G (previously denoted as p.C1039G) of the *fibrillin1* gene (*Fbn1*) identified from human patients with Marfan syndrome^14–16^. As TAA is a major life-threatening complication of Marfan syndrome, *Fbn1* KI mice serve as a good model for TAA. The other model is the β-aminopropionitrile (BAPN)-treated mice. BAPN is an inhibitor of lysyl oxidase (LOX), which mediates the crosslinking process of elastin and collagen^17^. TAA and thoracic aortic dissection were successfully induced in 3-week-old mice fed directly with BAPN for 4 weeks^17^.

TGF-β1 plays an important role in a plethora of physiological and pathophysiological processes (e.g., organismal aging, development, cellular senescence, immune response, inflammation, and tissue fibrosis) and in the pathogenesis of many diseases, including aneurysms, hypertension, atherosclerosis, and cancer^18^. The activation of TGF-β1 requires cleavage of an amino-terminal fragment, called the latency-associated peptide (LAP) from its precursor LAP-TGF-β1^19^. Activation of latent TGF-β1 is the most important step in the regulation of TGF-β function, and some known mechanisms include proteolysis by plasmin, binding to integrin αV, oxidative modifications, and interaction with thrombospondin 1^19^. We recently showed that ADAMTS16 interacts with LAP-TGF-β1 and activates TGF-β1^19^.

*AGGF1* was originally discovered by us as a gene for vascular disease Klippel-Trenaunay syndrome (KTS)^20^. *AGGF1* encodes an angiogenic factor that promotes angiogenesis as potentially as VEGFA^20^, and is required for vasculogenesis and angiogenesis in vivo^21^. However, in contrast to VEGFA that promotes vascular permeability, AGGF1 blocks vascular permeability, and thereby becomes a better agent for therapeutic angiogenesis^22^. AGGF1 is a master regulator for cell proliferation, migration, and differentiation, and acts at the top of the genetic regulatory system that promotes the differentiation of mesodermal cells into pluripotent hemangioblasts in zebrafish^23,24^. Recently, we identified the receptor for AGGF1 as integrin α5 on endothelial cells (ECs)^25^, and α7 on vascular smooth muscle cells (VSMCs)^26^. AGGF1 is also a key regulator for autophagy, ER stress, p53 stability, and alternative splicing^22,27^.

Considering the important role of AGGF1 in VSMC functions, we investigated the role of AGGF1 in vascular remodeling associated with TAA. Using TAC as a model for arterial remodeling associated with TAA in response to increased biomechanical forces^11^, we demonstrated that AGGF1 is an important molecular determinant of vascular inflammation and remodeling, and AGGF1 protein therapy can successfully inhibit vascular inflammation and remodeling associated with TAA. The conclusions from the TAC model were validated in a genetic model for TAA (*Fbn1*^*C1041G/+*^ mice) and a BAPN-induced TAA model. Mechanistically, AGGF1 interacts with its receptor integrin α7, and enhances the interaction between integrin α7 and LAP-TGF-β1, blocking maturation of TGF-β1 and TGF-β1 signaling as well as ERK1/2 signaling.

## Results

### AGGF1 expression is reduced in thoracic aortic tissue from TAA patients

Immunostaining was performed for AGGF1 using sections of ascending aortas from eight TAA patients and eight non-TAA controls. The results showed that the AGGF1 level was markedly reduced in TAA patients compared with non-TAA controls (Supplementary Fig. S1).

### ***Aggf1*haploinsufficiency aggravates pressure-overload-induced vascular inflammation and remodeling**

To further study the reduced expression of AGGF1 by vascular remodeling as in TAA patients, we studied TAC mice, a pressure-overload model for aortic aneurysms. We performed TAC first for the right common carotid arteries, and used the left common carotid arteries as a normotension control. At the 21st day after TAC, Doppler echocardiography analysis demonstrated that the ratio of the velocity of blood flow in the right carotid artery over that in the left carotid artery from TAC mice was 4-fold faster than that from control sham-operated mice (Supplementary Fig. S2a). The diameter of the right carotid artery (RCA) was increased by 35%, and the artery was thickened in TAC mice compared with sham-operated mice (Supplementary Fig. S2b–c). The cell density (the total number of cells divided by the area) in adventitia, but not the cell density in the medial layer, was increased by 3-fold in TAC mice compared with sham-operated mice (Supplementary Fig. S2c). These characteristics are similar to vascular remodeling from ANG II-induced ascending aortic aneurysms^28^. Consistent with the data from human TAA patients, both immunostaining and Western blot analyses showed that the expression of AGGF1 in carotid arteries was dramatically decreased three weeks after TAC in mice compared to age-matched sham-operated mice (Supplementary Fig. S2d–e).

**Fig. 1:**
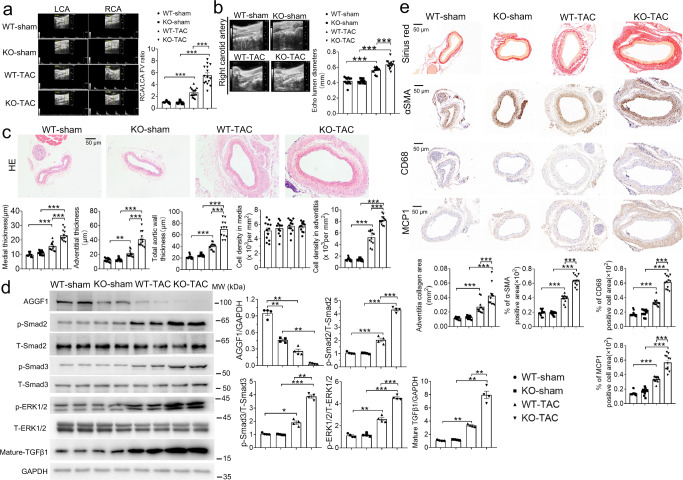
*Aggf1* haploinsufficiency aggravates TAC-induced arterial dilatation, remodeling, and inflammation in male mice. **a** Echocardiographic images of LCA and RCA from *Aggf1*^*+/+*^ (WT) or *Aggf1*^*+/−*^ (KO) mice three weeks after TAC or sham operation. The bar graph on the right shows the ratio of blood flow velocity (FV) of RCA over LCA (*n* = 12, 12, 15, and 15 mice). **b** Mean lumen diameter of RCA from WT or KO mice (*n* = 12, 12, 15, and 15 mice). **c** H&E staining of cross-sections of RCA (*n* = 12 mice/group). Bar graphs show medial thickness (1st graph), adventitial thickness (2nd graph), total aortic wall thickness (3rd graph), cell density in media (4th graph), and cell density in adventitia (5th graph). **d** Western blot analysis was carried out for AGGF1, phosphorylated ERK1/2 (p-ERK1/2), total ERK1/2 (T-ERK1/2), phosphorylated Smad2 (p-Smad2), total Smad2 (T-Smad2), phosphorylated Smad3 (p-Smad3), total Smad3 (T-Smad3), and mature TGF-β1. GAPDH was used as loading control. Quantitative data are graphed on the right (*n* = 4 experiments/group). **e** Sirius red staining and immunostaining for α-smooth muscle actin (α-SMA) for smooth muscle cells, inflammation marker MCP-1 and macrophage marker CD68 with cross-sections of RCA. The bar graphs at the bottom show quantification of the collagen area, and the percentage of α-SMA-positive areas, MCP1 positive cell areas, and CD68 positive cell areas (*n* = 12 experiments/group). *P < 0.05; **P < 0.01; ***P < 0.001; Quantitative data were shown as mean ± SEM. Statistical analysis was performed with two-way ANOVA with Tukey’s tests (**a**–**e**).

Doppler echocardiography showed that after TAC, heterozygous *Aggf1*^+/−^ KO mice (i.e., *Aggf1* haploinsufficiency) showed a significantly increased flow velocity (FV) ratio of RCA over the unimpacted left carotid artery (LCA) and RCA dilatation compared with wild type (WT) *Aggf1*^+/+^ mice (Fig. 1a, b and Supplementary Fig. S3a–b). H&E staining showed that the carotid artery wall thickening, medial hypertrophy, adventitial hypertrophy, and cellular hyperplasia were more severe in *Aggf1*^+/−^-TAC mice than in *Aggf1*^+/+^-TAC mice (Fig. 1c and Supplementary Fig. S3c). Similar results were obtained from male and female mice (Fig. 1a–c and Supplementary Fig. S3). Thus, *Aggf1* haploinsufficiency exacerbates vascular remodeling in TAC mice.

To identify the molecular mechanisms by which *Aggf1* haploinsufficiency exacerbates vascular remodeling, we examined TGF-β1 signaling (phosphorylation of Smad2/3, level of mature TGF-β1), ERK1/2 activation, and vascular inflammation. As shown in Fig. 1d and Supplementary Fig. S4b, the level of mature TGF-β1 and phosphorylation levels of Smad2/3 and ERK1/2 were significantly increased after TAC in both *Aggf1*^+/−^ and *Aggf1*^+/+^ mice. However, compared to *Aggf1*^+/+^-TAC mice, the level of mature TGF-β1 and phosphorylation levels of Smad2/3 and ERK1/2 were further significantly increased in *Aggf1*^+/−^-TAC mice (Fig. 1d and Supplementary Fig. S4b). Sirius red staining showed that the collagen level in the adventitia of *Aggf1*^+/−^-TAC mice was significantly higher than that of *Aggf1*^+/+^-TAC mice, and the number of cells expressing α-SMA (VSMC proliferation) in arteries also increased significantly (Fig. 1e and Supplementary Fig. S4a). Immunostaining analysis showed that the signal intensity for MCP-1 (marker for inflammation) and CD68 (marker for macrophage infiltration) was dramatically increased in *Aggf1*^+/−^-TAC mice compared to *Aggf1*^+/+^-TAC mice (Fig. 1e and Supplementary Fig. S4a). Similar results were obtained from male and female mice (Fig. 1d–e and Supplementary Fig. S4). These data suggest that *Aggf1* haploinsufficiency exacerbates vascular remodeling by inducing TGF-β1 and ERK1/2 signaling and vascular inflammation.

### SMC-specific ***Aggf1*** knockout (KO) exacerbates pressure-overload-induced vascular inflammation and remodeling

**Fig. 2:**
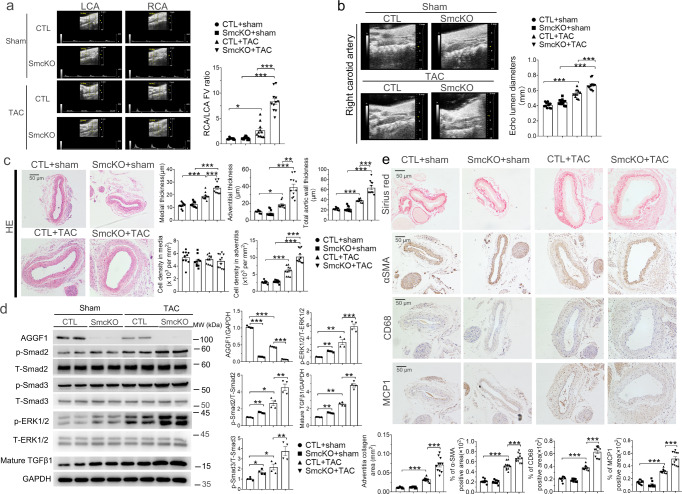
VSMC KO of *Aggf1* aggravates TAC-induced arterial dilatation, remodeling, and inflammation in male mice. **a** Echocardiographic images of LCA and RCA from *Aggf1*^*fl/fl*^ and *Aggf1*^*smcKO*^ mice three weeks after TAC or sham operation. The bar graph on the right shows the ratio of flow velocity (FV) of RCA over LCA (*n* = 12, 12, 11, and 11 mice). **b** Mean lumen diameter of RCA (*n* = 11, 12, 11, and 11 mice). **c** H&E staining of cross-sections of RCA, and quantification data on medial thickness (1st graph), adventitial thickness (2nd graph), total aortic wall thickness (3rd graph), cell density in media (4th graph), and cell density in adventitia (5th graph) (*n* = 10, 10, 11, and 11 mice). **d** Western blot analysis for AGGF1, p-ERK1/2, T-ERK1/2, p-Smad2, T-Smad2, p-Smad3, T-Smad3, and mature TGF-β1 of RCA. GAPDH was used as loading control (*n* = 4 experiments/group). **e** Sirius red staining and immunostaining for α-SMA, MCP-1, and CD68 with cross-sections of RCA. The bar graphs at the bottom show quantification of the collagen area, and the percentage of α-SMA/CD68/MCP1 positive areas (*n* = 10, 10, 11, and 11 mice). *P < 0.05; **P < 0.01; ***P < 0.001; Quantitative data were shown as mean ± SEM. Statistical analysis was performed with two-way ANOVA with Tukey’s tests (**a**–**e**).

*Aggf1* flox mice (*Aggf1*^*fl/fl*^; CTL) were bred to *Tagln-Cre* transgenic mice to generate smooth muscle cell (SMC)-specific *Aggf1* KO mice (*Aggf1*^*smcKO*^; smcKO) (Supplementary Fig. S5a). Western blotting showed that compared with CTL mice, smcKO mice showed barely detectable AGGF1 expression in the blood vessels (Supplementary Fig. S5b), but no difference was found in the heart, kidney, lung or liver between the two groups of mice (Supplementary Fig. S5c). Echocardiography showed that 21 days after TAC, the RCA/LCA FV ratio and lumen diameter of smcKO mice were significantly increased compared with that of CTL mice (Fig. 2a, b and Supplementary Fig. S6a–b). Similar to *Aggf1*^*+/-*^ mice, smcKO mice showed artery wall thickening, medial hypertrophy, adventitial hypertrophy, and cellular hyperplasia (Fig. 2c and Supplementary Fig. S6c). The level of mature TGF-β1 and phosphorylation levels of Smad2/3 and ERK1/2 were significantly increased in smcKO mice compared with CTL mice, and the effects were exacerbated after TAC (Fig. 2d and Supplementary Fig. S8). Moreover, smcKO mice showed increased collagen disposition, increased SMC density, and increased vascular inflammation (MCP-1 and CD68) (Fig. 2e and Supplementary Fig. S7). Similar results were obtained from male and female mice (Fig. 2 and Supplementary Figs. S6–8). These data suggest that VSMC-specific *Aggf1* KO exacerbates vascular remodeling by inducing TGF-β1 and ERK1/2 signaling and vascular inflammation.

### Recombinant AGGF1 protein attenuates vascular inflammation and remodeling by inhibiting TGF-β1 and ERK1/2 signaling in RCA

**Fig. 3:**
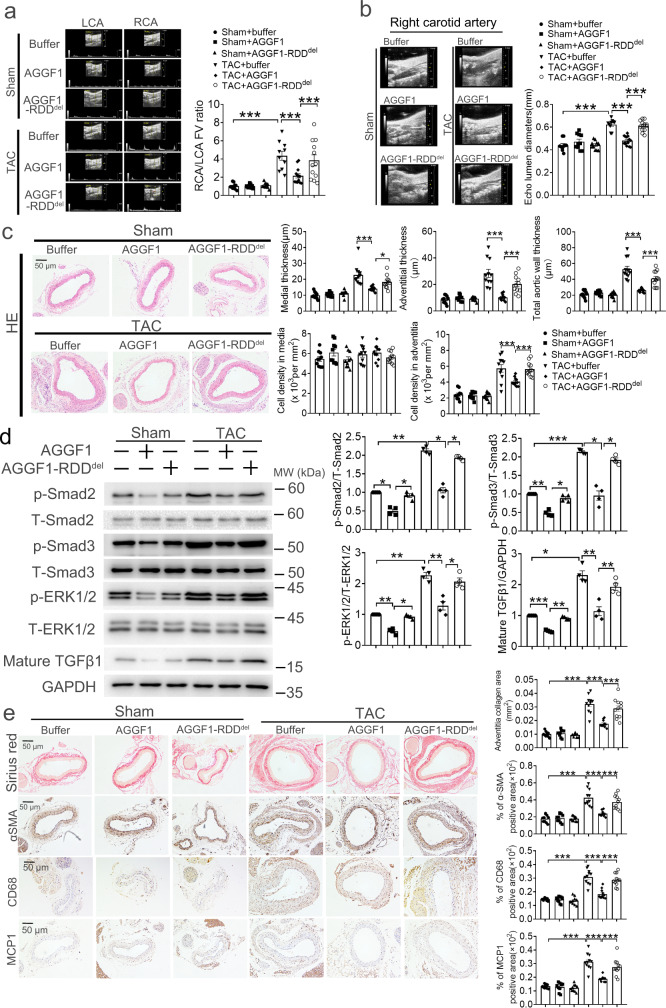
Intraperitoneal injection of purified human AGGF1 protein attenuates TAC-induced arterial dilatation and remodeling, vascular inflammation, TGF-β1 maturation and signaling, and ERK1/2 signaling in male mice. **a** Echocardiographic images of LCA and RCA from mice three weeks after TAC or sham operation and with or without wild-type AGGF1 or mutant AGGF1-RDD^del^ treatment. The bar graph on the right shows the ratio of flow velocity (FV) of RCA over LCA (*n* = 10, 10, 10, 10, 13, and 12 mice). **b** Mean lumen diameter of RCA (*n* = 10, 10, 10, 10, 13, and 12 mice). **c** H&E staining of cross-sections of RCA. Bar graphs at the right show medial thickness (1st graph), adventitial thickness (2nd graph), total aortic wall thickness (3rd graph), cell density in media (4th graph), cell density in adventitia (5th graph) (*n* = 10, 10, 10, 10, 11, and 11 mice). **d** Western blot analysis for p-ERK1/2, T-ERK1/2, p-Smad2, T-Smad2, p-Smad3, T-Smad3, and mature TGF-β1 of RCA. GAPDH was used as loading control (n = 4 experiments/group). **e** Sirius red staining and immunostaining for α-SMA, MCP-1 and CD68 with cross-sections of RCA. The bar graphs at the right show quantification of the collagen area, and the percentage of α-SMA/CD68/MCP1 positive areas (*n* = 10, 10, 10, 10, 11, and 11 mice). *P < 0.05; **P < 0.01; ***P < 0.001; Quantitative data were shown as mean ± SEM. Statistical analysis was performed with two-way ANOVA with Tukey’s tests (**a**–**e**).

To develop a pharmacologic therapy for aortic aneurysms, we examined the effects of AGGF1 protein therapy. We started to treat the TAC mice with intraperitoneal injection of the purified AGGF1 protein one day after the TAC procedure, and continued the treatment once every two days for three weeks. AGGF1 normalized typical characteristics of TAC-induced vascular remodeling in RCA (Fig. 3a–c and Supplementary Fig. S9). In addition, AGGF1 also normalized TAC-induced increases of mature TGF-β1 and phosphorylation levels of Smad2/3 and ERK1/2, and vascular inflammation (increased MCP-1 and CD68 signals) (Fig. 3d, e and Supplementary Fig. S10). Interestingly, AGGF1 also inhibited the level of mature TGFβ1 and phosphorylation of ERK1/2 and Smad2/3 from sham mice (Fig. 3d and Supplementary Fig. S10b). Similar results were obtained from male and female mice (Fig. 3 and Supplementary Figs. S9–10). All these data suggest that AGGF1 protein therapy can successfully attenuate pressure-overload-induced carotid artery dilatation, remodeling and inflammation, and VSMC proliferation by inhibiting TGF-β1 signaling and ERK1/2 signaling.

We recently identified the AGGF1 receptor on VSMCs as integrin α7, and the AGGF1-receptor interaction involved the RDD motif at amino acid positions 606-608 in the AGGF1 protein and between the FHA domain and the G-patch domain^26^. The function of AGGF1 was impaired if the RDD residues were deleted (referred to as AGGF1-RDD^del^)^26^. In TAC-induced vascular remodeling model, all effects of wild-type AGGF1 were almost abolished by AGGF1-RDD^del^ (Fig. 3 and Supplementary Figs. S9–10). The data suggest that the interaction domain between AGGF1 and integrin α7 is essential for the effects of AGGF1 on TGF-β1 signaling, ERK1/2 signaling, vascular inflammation, and remodeling.

### AGGF1 attenuates vascular inflammation and remodeling by inhibiting TGF-β1 and ERK1/2 signaling in the ascending aortas of TAC mice

To examine vascular modeling in TAA more closely, we examined the effects of AGGF1 on the ascending aortas from male TAC mice. As shown in Supplementary Figs. S11–12, compared with sham mice, TAC mice showed significantly increased aortic dilatation, medial hypertrophy, adventitial hypertrophy, cellular hyperplasia, increased collagen disposition, increased VSMC proliferation, and increased vascular inflammation (MCP-1 and CD68) in the ascending aortas. All these effects were attenuated by intraperitoneal injection of WT AGGF1, but not by mutant AGGF1-RDD^del^ (Supplementary Figs. S11–12). Western blot analysis showed that ERK1/2 and Smad2/3 phosphorylation and the amount of mature TGF-β1 in the ascending aortas were significantly elevated after TAC, and these effects were suppressed by WT AGGF1, but not by mutant AGGF1-RDD^del^ (Supplementary Fig. S11c). Similar results were obtained from the ascending aortas of female TAC mice (Supplementary Figs. S13–14). The data indicate that similar to RCA, AGGF1 can attenuate pressure-overload-induced arterial dilatation, remodeling and inflammation, and VSMC proliferation by inhibiting TGF-β1 signaling and ERK1/2 signaling in the ascending aortas.

### AGGF1 blocks TGF-β1 signaling, ERK1/2 signaling, and inflammation in VSMCs

**Fig. 4:**
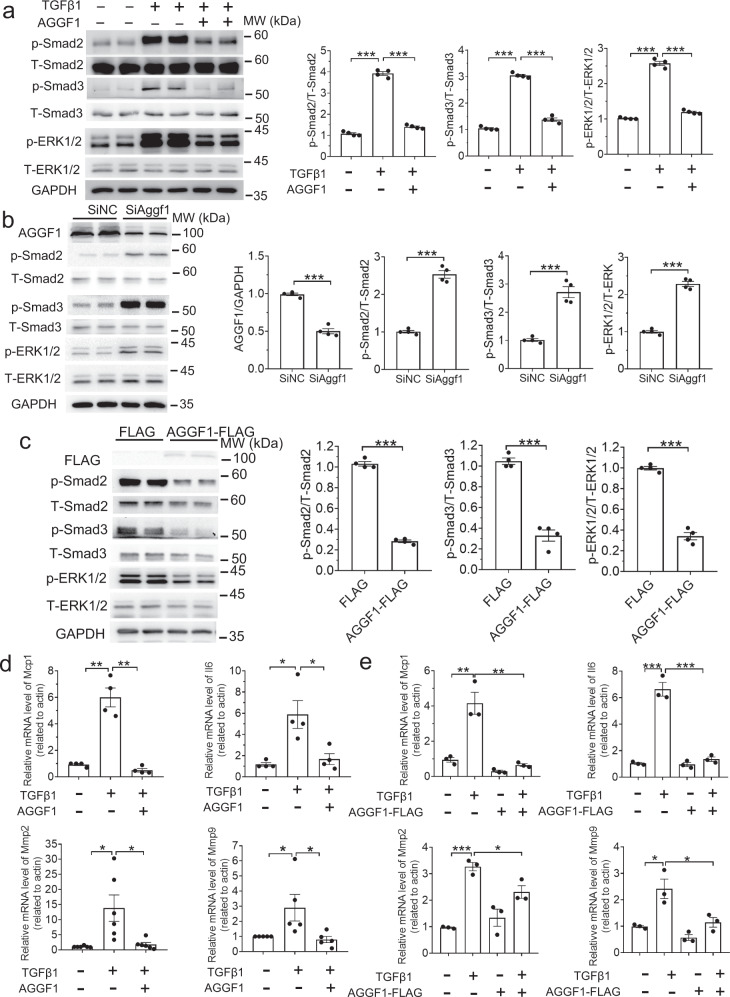
AGGF1 inhibits TGF-β1 and ERK1/2 signaling and TGF-β1 induced inflammation in VSMCs. **a** Western blot analysis for p-ERK1/2, T-ERK1/2, p-Smad2, T-Smad2, p-Smad3, and T-Smad3 using VSMCs starved for 12 h, treated with or without TGF-β1 for 15 minutes, and then treated with AGGF1 for 10 min. GAPDH was used as loading control (*n* = 4 experiments/group). **b** Western blot analysis using VSMCs transected with siRNA for *Aggf1* (SiAggf1) or negative control siRNA (SiNC) (*n* = 4 experiments/group). **c** Western blot analysis using VSMCs with or without over-expression of AGGF1-FLAG. GAPDH was used as loading control (*n* = 4 experiments/group). **d** Real-time RT-PCR analysis for measuring the mRNA level of *Il-6*, *Mcp1*, *Mmp2* and *Mmp9* using VSMCs treated with TGF-β1 for 24 h, then treated with AGGF1 for 24 hours. Gene expression levels were normalized to *β-actin* (*n* = 4, 4, 6, and 5 experiments/group for *Mcp1/Il6/Mmp2/Mmp9*, respectively). **e** Real-time RT-PCR analysis for *IL-6*, *Mcp1*, *Mmp2* and *Mmp9* using VSMCs with or without over-expression of AGGF1-FLAG, then treated with TGF-β1 for 48 h. Gene expression levels were normalized to *β-actin* (n = 3 experiments/group). *P < 0.05; **P < 0.01; ***P < 0.001; Quantitative data were shown as mean ± SEM. Statistical analysis was performed with two-way ANOVA with Tukey’s tests (**a**, **d**, **e**) or two-tailed unpaired Student’s *t*-tests (**b**, **c**).

To substantiate the in vivo findings in *Aggf1*^*+/−*^ mice and smcKO mice, we characterized the effects of AGGF1 on TGF-β1 signaling in cultured VSMCs. We treated VSMCs with different concentrations of TGF-β1 and found that TGF-β1 induced phosphorylation of Smad2/3 and ERK1/2 in a concentration-dependent and time-dependent manner (Supplementary Fig. S15a–b). The AGGF1 protein treatment eliminated the TGF-β1-induced increases of phosphorylated ERK1/2 and Smad2/3 in VSMCs (Fig. 4a). Moreover, AGGF1 inhibited the phosphorylation of ERK1/2 and Smad2/3 in the absence of exogenous TGF-β1 in a concentration-dependent manner (Supplementary Fig. S15c). Knockdown of *Aggf1* expression with siRNA significantly enhanced the phosphorylation levels of Smad2/3 and ERK1/2, while over-expression of AGGF1 inhibited the phosphorylation of Smad2/3 and ERK1/2 (Fig. 4b, c). These data indicate that AGGF1 inhibits TGF-β1 signaling in VSMCs, which validates the in vivo findings from mice.

TGF-β1 significantly increased the expression of inflammation-related genes, including *Il-6*, *Mcp-1*, *Mmp2*, and *Mmp9* in VSMCs (Fig. 4d, e). However, TGF-β1-induced upregulation of inflammation-related genes was blocked by the addition of AGGF1 into the culture media (Fig. 4d, e).

### AGGF1 blocks TGF-β1 signaling by inhibiting the maturation of TGF-β1 from LAP-TGF-β1

**Fig. 5:**
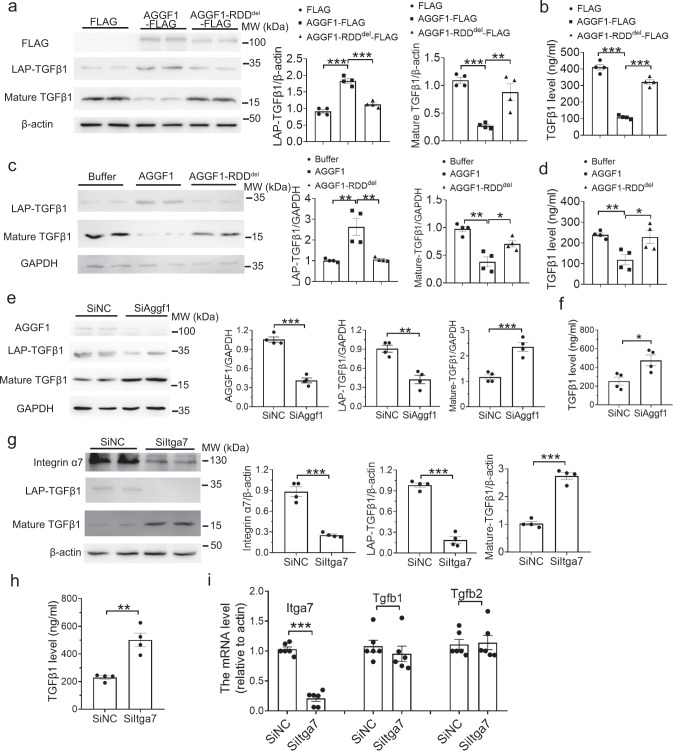
AGGF1 inhibits TGF-β1 maturation and knockdown of *Aggf1* or *Itga7* induces TGF-β1 maturation. **a** Western blot for LAP-TGF-β1 and mature TGF-β1 using VSMCs with overexpression of wild-type AGGF1 or mutant AGGF1-RDD^del^. β-actin was used as loading control (*n* = 4 experiments/group). **b** ELISA for measurement of TGF-β1 in culture media from VSMCs with overexpression of AGGF1 or AGGF1-RDD^del^ (*n* = 4 experiments/group). **c** Western blot for LAP-TGF-β1 and mature TGF-β1 using VSMCs treated with AGGF1 or AGGF1-RDD^del^ for 48 hours. GAPDH was used as loading control (*n* = 4 experiments/group). **d** ELISA for measurement of TGF-β1 in culture media from VSMCs treated with AGGF1 or AGGF1-RDD^del^ for 48 h (*n* = 4 experiments/group). **e** Western blot for LAP-TGF-β1 and mature TGF-β1 using VSMCs transfected with SiAggf1 or SiNC (*n* = 4 experiments/group). **f** ELISA for measurement of TGF-β1 in culture media from VSMCs transfected with SiAGGF1 or SiNC (*n* = 4 experiments/group). **g** Western blot for integrin α7, LAP-TGF-β1 and mature TGF-β1 using VSMCs transfected with siRNA for *Itga7* (SiItga7) or SiNC (*n* = 4 experiments/group). **h** ELISA for measurement of TGF-β1 in culture media from VSMCs transfected with SiItga7 or SiNC (*n* = 4 experiments/group). **i** Real-time RT-PCR analysis for *Itga7*, *Tgfb1* and *Tgfb2* using VSMCs transfected with SiItga7 or SiNC (*n* = 6 experiments/group). *P < 0.05; **P < 0.01; ***P < 0.001; *n* ≥ 3/group for studies. Quantitative data were shown as mean ± SEM. Statistical analysis was performed with two-way ANOVA with Tukey’s tests (**a**–**d**) or two-tailed unpaired Student’s *t*-tests (**e**–**h**).

To identify the molecular mechanism by which AGGF1 blocks TGF-β1 signaling, we examined the maturation of TGF-β1, i.e., the furin cleavage of LAP-TGF-β1 into LAP and mature TGF-β1, an essential step in consequent activation of TGF-β1^19^. AGGF1 protein treatment significantly increased LAP-TGF-β1, and decreased mature TGF-β1 and secreted TGF-β1, whereas mutant AGGF1-RDD^del^ lost all these effects (Fig. 5a–d). AGGF1 did not affect the expression of *Tgfb1* and *Tgfb2* mRNA (Supplementary Fig. S16a, b). Knockdown of *Aggf1* expression with siRNA significantly decreased LAP-TGF-β1, and increased mature TGF-β1 and secreted TGF-β1 (Fig. 5e, f), but did not have any effect on the expression of *Tgfb1* and *Tgfb2* mRNA (Supplementary Fig. S16c).

We also investigated whether integrin α7, the AGGF1 receptor on VSMCs, played a role in TGF-β1 maturation. Similar to knockdown of *Aggf1*, knockdown of *Itga7* using siRNA significantly decreased LAP-TGF-β1 (Fig. 5g), and increased mature TGF-β1 (Fig. 5g) and secreted TGF-β1 (Fig. 5h), but did not have any effect on the expression of *Tgfb1* and *Tgfb2* mRNA (Fig. 5i).

**Fig. 6:**
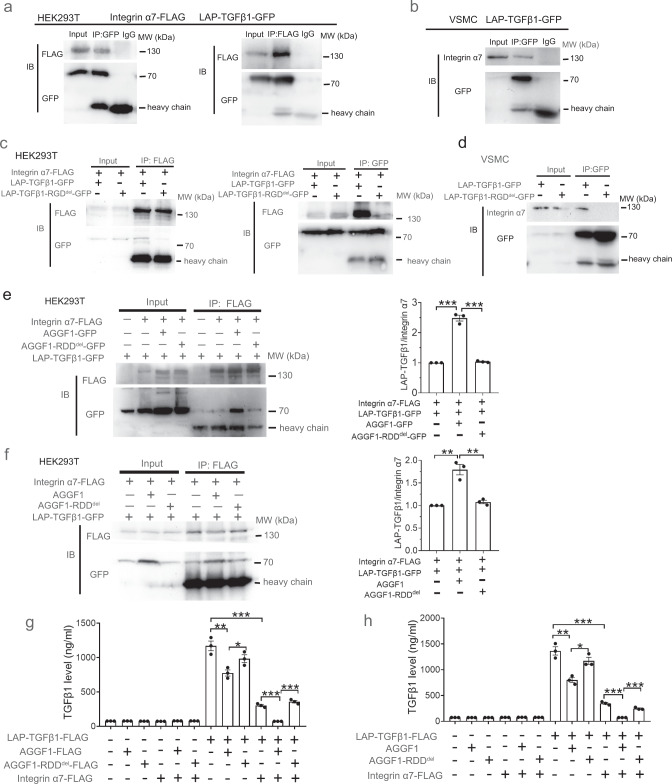
Integrin α7 interacts with LAP-TGF-β1 through the RGD sequence and AGGF1 enhances the interaction. **a** Co-IP using HEK293T cells with overexpression of LAP-TGF-β1-GFP and integrin α7-FLAG. **b** Co-IP for LAP-TGF-β1 and endogenous integrin α7 using VSMCs with overexpression of LAP-TGF-β1-GFP. **c** Co-IP using HEK293T with overexpression of wild-type LAP-TGF-β1-GFP or mutant LAP-TGF-β1-RGD^del^-GFP together with integrin α7-FLAG. **d** Co-IP using VSMCs with overexpression of LAP-TGF-β1-GFP or LAP-TGF-β1-RGD^del^-GFP. **e** Co-IP using HEK293T with overexpression of LAP-TGF-β1-GFP, α7-FLAG, wild- type AGGF1-GFP or mutant AGGF1-RDD^del^-GFP. The quantified data of the ratios of LAP-TGF-β1-GFP/α7-FLAG are plotted on the right. **f** Co-IP using HEK293T with overexpression of LAP-TGF-β1-GFP and integrin α7-FLAG, and stimulated with AGGF1 or AGGF1-RDD^del^ for 24 h. The quantified data of the ratios of LAP-TGF-β1-GFP/α7-FLAG are plotted on the right. **g** ELISA analysis for measurement of TGF-β1 in culture media from HEK293T cells transfected with different plasmids for LAP-TGF-β1-FLAG, integrin α7-FLAG, AGGF1-FLAG or AGGF1-RDD^del^-FLAG. **h** ELISA analysis for measurement of TGF-β1 in culture media from HEK293T cells transfected with different plasmids for LAP-TGF-β1-FLAG and integrin α7-FLAG, and treated with purified AGGF1 or AGGF1-RDD^del^ for 24 h. *P < 0.05; **P < 0.01; ***P < 0.001; Quantitative data were shown as mean ± SEM. *n* = 3 experiments/group. Statistical analysis was performed with two-way ANOVA with Tukey’s tests (**e**–**h**).

To further identify the molecular mechanism by which AGGF1 blocks TGF-β1 maturation, we investigated whether integrin α7 interacts with TGF-β1 and the impact of AGGF1 on the interaction. We used HEK293T cells with co-expression of α7-FLAG and LAP-TGF-β1-GFP for Co-IP analysis and found that integrin α7 and LAP-TGF-β1 strongly immunoprecipitated each other, demonstrating the interaction between the two proteins (Fig. 6a). Further Co-IP analysis using VSMCs with overexpression of only LAP-TGF-β1-GFP showed that LAP-TGF-β1 interacted with endogenous integrin α7 in VSMCs (Fig. 6b). Mutations in the RGD motif of LAP was shown to disrupt the interaction between LAP-TGF-β1 and integrin αV^29^. Similarly, we created a mutant LAP-TGF-β1 with the LAP RGD motif deleted (LAP-TGF-β1-RGD^Del^). Co-IP analysis showed that LAP-TGF-β1-RGD^Del^ showed reduced affinity to integrin α7 in both HEK293T cells and VSMCs (Fig. 6c, d). We also examined the interaction between AGGF1 endothelial cell receptor integrin α5 and LAP-TGF-β1 and found that there was no interaction between integrin α5 and LAP-TGF-β1 (Supplementary Fig. S17). The data suggest that LAP-TGF-β1 interacts with the AGGF1 receptor on VSMCs (integrin α7) but not that on endothelial cells (integrin α5).

Interestingly, Co-IP analysis showed that co-expression of AGGF-GFP or AGGF1 enhanced the interaction between integrin α7 and LAP-TGF-β1, however, mutant AGGF1-RDD^del^ did not show such an effect (Fig. 6e, f). Functionally, ELISA analysis showed that the level of secreted TGF-β1 in culture media was low in HEK293T cells, but dramatically increased in HEK293T cells with overexpression of TGF-β1 (Fig. 6g, h). Co-expression of AGGF1-FLAG, but not mutant AGGF1-RDD^del^-FLAG, inhibited the maturation of TGF-β1 and significantly reduced the level of secreted TGF-β1 (Fig. 6g). Co-expression of integrin α7 inhibited the maturation of TGF-β1 and dramatically reduced the level of secreted TGF-β1, and this effect was markedly strengthened by co-expression of AGGF1-FLAG, but not AGGF1-RDD^del^ (Fig. 6g). Similar results were obtained with cells treated with purified WT AGGF1, but not with mutant AGGF1-RDD^del^ (Fig. 6h).

### TGF-β1 inhibits AGGF1 expression

We analyzed the effect of TGF-β1 on AGGF1. We treated VSMCs with TGF-β1 for different time points (2 and 4 days). Western blot analysis showed that at both time points, TGF-β1 significantly reduced the protein level of AGGF1 (Supplementary Fig. S18a). We then treated VSMCs with pirfenidone (PFD), a TGF-β inhibitor that inhibits the activity of the proteolytic enzyme furin that cleaves LAP-TGF-β1 into LAP and TGF-β1. Pirfenidone increased the level of LAP-TGF-β1, inhibited the maturation of TGF-β1 (Supplementary Fig. S18b), and reduced the secreted TGF-β1 level in media (Supplementary Fig. S18c). Pirfenidone significantly increased the expression of AGGF1 protein at day 4, but not at day 2 (Supplementary Fig. S18d). Pirfenidone did not have any significant effect on the mRNA expression of *Tgfb*1 and *Tgfb2* (Supplementary Fig. S18e).

### Functional effect of pirfenidone (PFD) is dependent on AGGF1

**Fig. 7:**
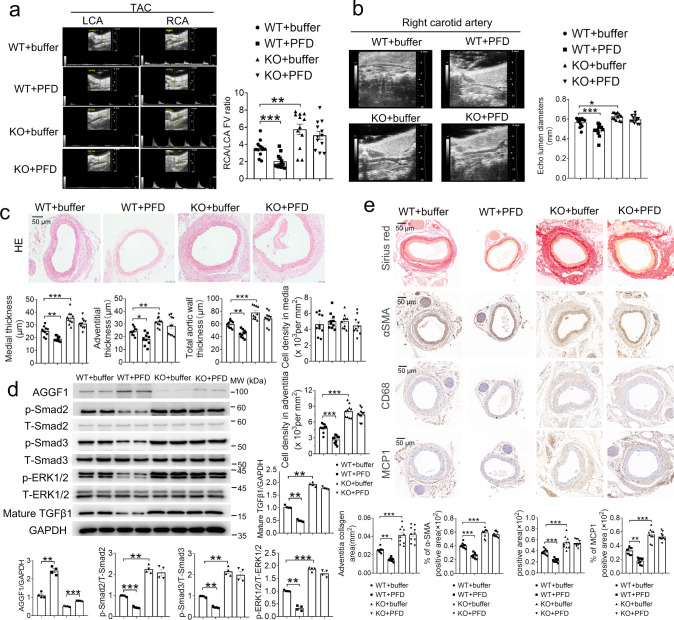
Pirfenidone attenuates TAC-induced arterial dilatation, remodeling and inflammation by inhibiting Smad2/3, ERK1/2 phosphorylation, and mature TGFβ1 in male *Aggf1*^*+/+*^ (WT) mice, but not in male *Aggf1*^*+/−*^ (KO) mice. **a** Echocardiographic images of LCA and RCA from WT or KO mice three weeks after TAC with or without pirfenidone treatment. The bar graph on the right shows the ratio of flow velocity (FV) of RCA over LCA (*n* = 12 mice/group). **b** Mean lumen diameter of RCA (*n* = 12/group). **c** H&E staining of cross-sections of RCA. Bar graphs show medial thickness, adventitial thickness, total aortic wall thickness, cell density in media, and cell density in adventitia (*n* = 10 mice/group). **d** Western blot analysis for AGGF1, p-ERK1/2, T-ERK1/2, p-Smad2, T-Smad2, p-Smad3, T-Smad3 and mature TGF-β1. GAPDH was used as loading control (*n* = 4 experiments/group). **e** Sirius red staining and immunostaining for α-SMA, MCP-1 and CD68 with cross-sections of RCA. The bar graphs on the bottom show quantification of the collagen area, and the percentage of α-SMA/CD68/MCP1 positive areas (*n* = 10 experiments/group). *P < 0.05; **P < 0.01; ***P < 0.001; Quantitative data were shown as mean ± SEM. Statistical analysis was performed with two-way ANOVA with Tukey’s tests (**a**–**e**).

As TGF-β1 inhibitor pirfenidone increased AGGF1 expression, we hypothesized that pirfenidone functions by relying on AGGF1. To test the hypothesis, we examined the effect of pirfenidone on vascular inflammation and modeling in TAC mice. Pirfenidone treatment significantly decreased the RCA/LCA FV ratio, attenuated right carotid artery dilatation, wall thickening, intimal hypertrophy, adventitia hypertrophy, and cell hyperplasia, reduced collagen deposition and VSMC proliferation, and inhibited vascular inflammation (MCP1 signal) and macrophage infiltration (CD68 signal) in WT *Aggf1*^+/+^-TAC mice, but not in *Aggf1*^+/−^-TAC mice (Fig. 7a–d and Supplementary Fig. S3–4). Western blot analysis showed that pirfenidone treatment significantly reduced mature TGF-β1 and the phosphorylation levels of Smad2/3 and ERK1/2 in the right carotid artery in *Aggf1*^+/+^-TAC mice, but not in *Aggf1*^+/−^-TAC mice (Fig. 7d and Supplementary Fig. S4b). The data indicate that the inhibitory effect of pirfenidone on TGF-β1 maturation and signaling depends on the presence of AGGF1, and suggest that therapeutic treatment with pirfenidone needs to consider to have a high expression level of AGGF1.

### Recombinant AGGF1 protein attenuates TAA in Fbn1^C1041G+^ mice

**Fig. 8:**
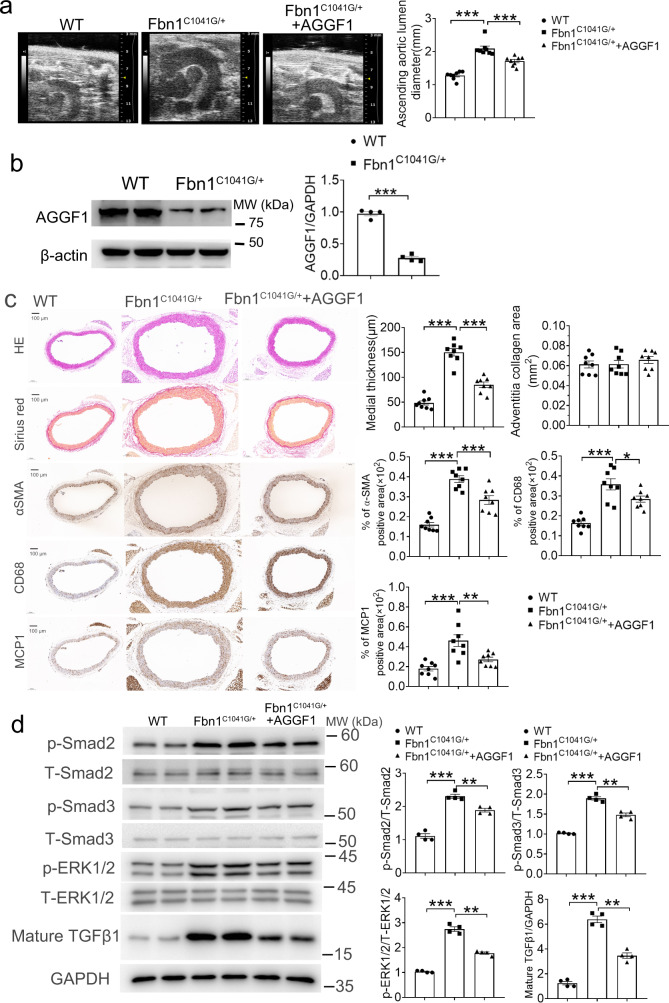
AGGF1 attenuates arterial remodeling, inflammation, and TGF-β1 signaling in a genetic model for TAA (male *Fbn1*^*C1041G/+*^ mice). **a** Mean lumen diameter of the ascending aortas from three groups of mice, including wild-type mice (WT), heterozygous *Fbn1*^*C1041G/+*^ mice treated with PBS buffer, and *Fbn1*^*C1041G/+*^ mice treated with recombinant AGGF1 protein. **b** Western blot analysis for AGGF1. β-actin was used as loading control. **c** H&E staining, Sirius red staining and immunostaining for α-SMA, MCP-1, and CD68 with cross-sections of ascending aortas. Bar graphs at the right show quantification of medial thickness, the collagen area, and the percentage of α-SMA/CD68/MCP1 positive areas. **d** Western blot analysis for p-ERK1/2, T-ERK1/2, p-Smad2, T-Smad2, p-Smad3, T-Smad3 and mature TGF-β1. GAPDH was used as loading control. *P < 0.05; **P < 0.01; ***P < 0.001; *n* = 8 experiments/group for mouse studies and *n* = 4/group for Western blot analysis. Quantitative data were shown as mean ± SEM. Statistical analysis was performed with two-way ANOVA with Tukey’s test (**a**, **c**, **d**) or two-tailed unpaired Student’s *t*-test (**b**).

To further examine the therapeutic effect of the AGGF1 protein on TAA, we studied *Fbn1*^*C1041G/+*^ mice, which are widely used as a genetic mouse model for TAA associated with Marfan syndrome^16^. Compared with wild-type mice, *Fbn1*^*C1041G/+*^ mice showed significantly increased aortic dilatation in the ascending aortas of both male and female mice (Fig. 8a and Supplementary Fig. S19a). Meanwhile, Western blot analysis showed that similar to human TAA patients, the expression level of AGGF1 was significantly reduced in the ascending aortas of *Fbn1*^*C1041G/+*^ mice (Fig. 8b). *Fbn1*^*C1041G/+*^ mice showed significantly increased medial hypertrophy, increased VSMC proliferation (α-SMA), and increased vascular inflammation (MCP-1 and CD68) (Fig. 8c and Supplementary Fig. S19b). Western blot analysis further showed that ERK1/2 and Smad2/3 phosphorylation levels and the amount of mature TGF-β1 in the ascending aortas were significantly elevated in *Fbn1*^*C1041G/+*^ mice. These TAA-associated abnormalities were attenuated by intraperitoneal injection of WT AGGF1 (Fig. 8d and Supplementary Fig. S19c). These data indicate that AGGF1 can reverse TAA abnormalities associated with Marfan syndrome by inhibiting the TGF-β1 maturation and signaling as well as ERK1/2 signaling.

### Recombinant AGGF1 protein attenuates aortic aneurysms in a BAPN-induced mouse model

**Fig. 9:**
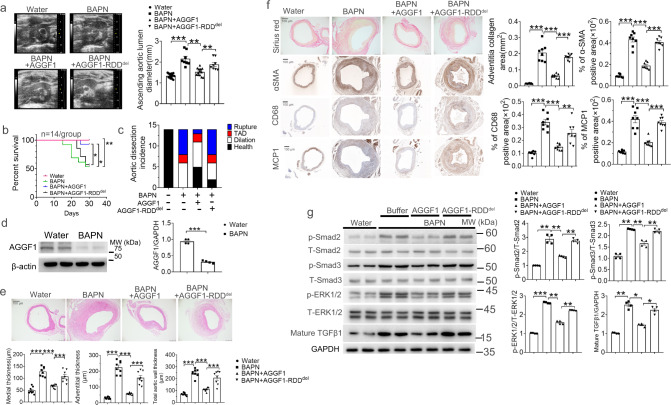
AGGF1 blocks arterial remodeling, inflammation, and TGF-β1 signaling in a BAPN-induced model for aortic aneurysms in male mice. **a** Mean lumen diameter of the ascending aortas from four groups of male mice (control mice fed with water, BAPN-fed mice treated with PBS buffer, BAPN-fed mice treated with wild type AGGF1, and BAPN-fed mice treated with mutant AGGF1 with a deletion of the RDD motif (*n* = 14, 8, 13, and 8 mice). **b** Kaplan-Meier survival curves and statistical analysis using a log-rank test. **c** Ratios of mice with thoracic aortic dissection (TAD), thoracic aortic rupture, and thoracic aortic dilatation among four different groups of mice. **d** Western blot analysis for AGGF1. β-actin was used as loading control (*n* = 4 experiments/group). **e**, **f** H&E staining, Sirius red staining and immunostaining for α-SMA, MCP-1 and CD68 with cross-sections of ascending aortas. Bar graphs at the right show quantification of medial thickness, adventitial thickness, total aortic wall thickness, the collagen area, and the percentage of α-SMA/CD68/MCP1 positive areas (*n* = 8 experiments/group). **g** Western blot analysis for p-ERK1/2, T-ERK1/2, p-Smad2, T-Smad2, p-Smad3, T-Smad3 and mature TGF-β1. GAPDH was used as loading control (*n* = 4 experiments/group). *P < 0.05; **P < 0.01; ***P < 0.001; Quantitative data were shown as mean ± SEM. Statistical analysis was performed with two-way ANOVA with Tukey’s tests (**a**, **e**, **f**, **g**), log-rank tests (**b**) or two-tailed unpaired Student’s *t*-tests (**d**).

BAPN is an interesting pharmacological agent that can induce TAA in 3-week-old mice when fed with 0.25% BAPN in water for 4 weeks^17^. Compared with mice fed with water alone, BAPN-fed mice developed the dilatation of ascending aortas by >1.5-fold (Fig. 9a and Supplementary Fig. S20a). Interestingly, the phenotype of ascending aortic dilatation in BAPN-treated mice was inhibited by the treatment with WT AGGF1 protein, but not with mutant AGGF1-RDD^del^ protein in both male and female mice (Fig. 9a and Supplementary Fig. S20a). The survival rate of BAPN-fed mice was much lower than the control water-fed mice in both male and female mice, however, the effect was blocked by the treatment with WT AGGF1 protein, but not by mutant AGGF1-RDD^del^ protein (Fig. 9b and Supplementary Fig. S20b). We also observed the development of more frequent thoracic aortic rupture and dissection (TAD) in BAPN-fed mice than in the control water-fed mice in both male and female mice, however, the effect was blocked by the treatment with WT AGGF1 protein, but not by mutant AGGF1-RDD^del^ protein (Fig. 9c and Supplementary Fig. S20c). Moreover, Western blot analysis showed that as with human TAA patients, the expression level of AGGF1 was significantly reduced in the ascending aortas of BAPN-treated mice (Fig. 9d).

Compared with control water-fed mice, BAPN-fed mice showed medial hypertrophy, adventitial hypertrophy, increased collagen disposition, increased VSMC proliferation, and increased vascular inflammation (MCP-1 and CD68) in the ascending aortas, however, the effect was blocked by the treatment with WT AGGF1 protein, but not by mutant AGGF1-RDD^del^ protein (Fig. 9e, f and Supplementary Fig. S20d, Supplementary Fig. S21a). Western blot analysis showed that ERK1/2 and Smad2/3 phosphorylation levels and the amount of mature TGF-β1 were significantly elevated in the ascending aortas of BAPN-fed mice, and these effects were inhibited by WT AGGF1, but not by mutant AGGF1-RDD^del^ (Fig. 9g and Supplementary Fig. S21b). Taken together, all these data indicate that AGGF1 protein therapy is effective in blocking the development of aortic aneurysms in the ascending aortas in the BAPN-induced mouse model.

## Discussion

The data in this study demonstrate that the AGGF1 protein therapy, i.e., the intraperitoneal injection of the purified recombinant AGGF1 protein, successfully attenuates TAA by blocking arterial dilatation and remodeling in three different mouse models, including a TAC-induced TAA model, a genetic TAA model, and a pharmacological BAPN-induced TAA model (Figs. 3, 8–9 and Supplementary Figs. S9–14, S19–21). The findings offer a potential treatment solution or a drug-target in our desperate searches for an effective pharmacological treatment for TAA. Surgical treatment remains the major intervention to treat TAA. Despite advances in the surgical treatments, TAA remain a major cause of morbidity and mortality, and many patients die before reaching a hospital for surgery. Moreover, surgical treatments have serious complications and adverse effects such as inflammation, thrombosis, aneurysm recurrence, and death^7,30^. To date, there is no effective therapeutic drug that can fully inhibit or reverse TAA. Habashi et al showed promising results for losartan, an AT1 receptor blocker used for treatment of hypertension, as a potential therapy to attenuate aortic dilatation in a mouse model for Marfan syndrome/TAA^15,31^. However, several clinical studies yielded inconsistent results about whether the effectiveness of losartan in attenuating aortic dilatation and aneurysm expansion is better than β-blockers^32,33^. The largest Pediatric Heart Network trial showed almost a doubling of adverse events with losartan treatment compared with β-blockers^33^. Taken together, these data emphasize the importance of our finding of an innovative protein therapy based on AGGF1 for TAA. Although the therapeutical potential of the AGGF1 protein for TAA was demonstrated only in the animal models, the promising data may encourage or facilitate the future clinical trials to assess its effectiveness in attenuating arterial dilatation and remodeling in human patients with TAA.

**Fig. 10:**
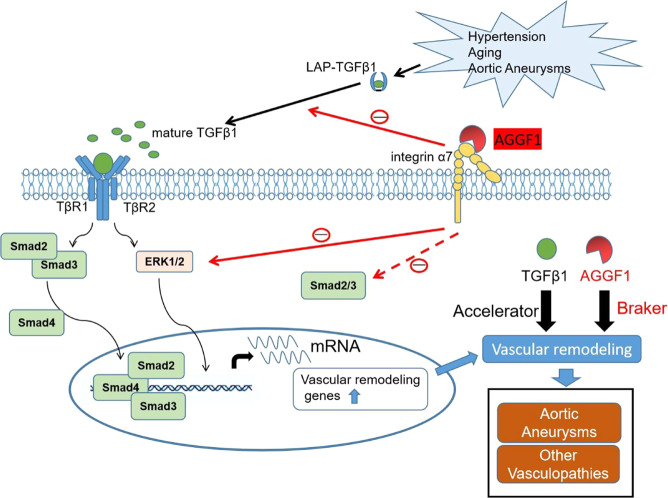
Molecular signaling pathways involved in vascular inflammation and remodeling. Pressure-overload as in case of hypertension induces cleavage of LAP-TGF-β1 and generation of mature TGF-β1, activates Smad phosphorylation and ERK1/2 signaling, and results in transcription of genes involved in vascular inflammation and remodeling. AGGF1 interacts with its receptor on VSMCs, integrin α7, and enhances the interaction between integrin α7 and TGF-β1, which inhibits cleavage of LAP-TGF-β1 and generation of mature TGF-β1, reduces Smad phosphorylation and ERK1/2 signaling, and results in reduced transcription of genes involved in vascular inflammation and remodeling, and attenuation of vasculopathies. TGF-β1 reduces AGGF1 expression in time- and concentration-dependent manners. Pirfenidone inhibits cleavage of LAP-TGF-β1 and generation of mature TGF-β1, reduces Smad phosphorylation and ERK1/2 signaling, and attenuates vascular inflammation and remodeling, but only when AGGF1 expression is high. TGF-β1 acts as an accelerator for vascular inflammation and remodeling, leading to development of vasculopathies such as aortic aneurysms, hypertension, and atherosclerosis. AGGF1 acts as a brake for vascular inflammation and remodeling, and inhibits the development of vasculopathies.

The number of people over 65 years is projected to double in the next 30 years^26^, and understanding aging is of critical importance in addressing age-related diseases. Aging-induced vascular remodeling is of particular importance as the vascular system is pervasive and plays an important role in every tissue in the body, and contributes to the pathogenesis of diseases such as cardiovascular and cerebrovascular diseases, vascular cognitive impairment, Alzheimer’s disease, and eye disease^26^. In this study, we established angiogenic factor AGGF1 as a molecular determinant of vascular inflammation and remodeling associated with TAA, and uncovered fundamental cellular and molecular mechanisms of vascular remodeling (Fig. 10). First, using TAC mice as a model for pressure-overload-induced vascular inflammation and remodeling associated with aortic aneurysms in both carotid arteries and ascending aortas in TAC mice, we showed that vascular remodeling significantly decreased the expression of AGGF1 (Supplementary Fig. S2). This important finding of AGGF1 downregulation in the ascending aortas of TAC mice was confirmed in the genetic TAA model, the BAPN-induced TAA model, and human patients with TAA (Supplementary Figs. S1, Fig. 8b and Fig. 9d). Most importantly, reduced AGGF1 expression in either heterozygous *Aggf1*^*+/-*^ mice or VSMC-specific *Aggf1* smcKO mice aggravated vascular inflammation and remodeling after TAC (Figs. 1–2 and Supplementary Figs. S3–8). The data suggest that the reduced AGGF1 expression is causative to TAA and AGGF1 therapy targets a fundamentally important molecular mechanism underlying TAA. Second, intraperitoneal injection of AGGF1 attenuated vascular inflammation and remodeling associated with TAA (Figs. 3, 8–9 and Supplementary Figs. S9–14, S19–21). Third, we found that AGGF1 enhanced the interaction between its VSMC receptor integrin α7 and LAP-TGF-β1, which led to inhibition of the maturation of TGF-β1 from LAP-TGF-β1, and reduced secretion of TGF-β1 (Figs. 4–6). This resulted in reduced TGF-β1 signaling (reduced Smad3 phosphorylation), reduced ERK1/2 signaling, and attenuation of vascular inflammation and remodeling (Fig. 10). We propose that TGF-β1 acts as an accelerator for vascular inflammation and remodeling, while AGGF1 acts as a natural antagonist of TGF-β1 to serve as a brake system to block vascular inflammation and remodeling associated with TAA (Fig. 10).

Our study showed that integrin α7 is the regulator of TGF-β1 maturation. Similar to knockdown of *Aggf1* expression, knockdown of *Itga7* significantly decreased LAP-TGF-β1, and increased mature TGF-β1 and secreted TGF-β1 (Fig. 5e–h). Co-expression of integrin α7 inhibited the maturation of TGF-β1, and this effect was markedly exacerbated by co-expression of AGGF1 (Fig. 6g, h). LAP-TGF-β1 interacted with integrin α7 through the RGD motif (Fig. 6c, d), and the interaction is the key for the maturation of TGF-β1 (Fig. 6g, h). In contrast, integrin α5, the AGGF1 receptor on endothelial cells, did not interact with TGF-β1 (Supplementary Fig. S17). Consistent with our finding, Turner et al showed that integrin α5β1 did not interact with LAP-TGF-β1, although it promoted TGF-β1 signaling^34^. Therefore, the interaction with LAP-TGF-β1 may be an important factor in distinguishing the different functions of AGGF1 in VSMCs and endothelial cells. Integrin αV is the only other integrin that was shown to bind to the RGD motif of LAP-TGF-β1 to promote the cleavage of LAP by furin^35^. Integrin α8β1 was shown to regulate TGF-β1-promoted cell adhesion, but did not interact with LAP-TGF-β1^35,36^. It is unknown why some integrins like αV and α7 bind to LAP-TGF-β1, but many others such as α5 and α8 do not interact with LAP-TGF-β1, however, it does suggest that the RGD motif is not sufficient for interaction with LAP-TGF-β1, and other sequences are needed. Together, these data suggest that integrin α7 is the second integrin that binds to LAP-TGF-β1, regulates cleavage of LAP-TGF-β, and controls TGF-β signaling. However, in contrast to αV that promotes TGF-β maturation, AGGF1 and integrin α7 inhibit TGF-β maturation (Fig. 5).

Interestingly, we found that TGF-β and AGGF1 formed a feedback regulatory loop. We showed that TGF-β inhibited the expression of AGGF1 on both the mRNA level and protein level in VSMCs, however, the effect was blocked by pirfenidone, a TGF-β precursor converting enzyme inhibitor (Supplementary Fig. S18). As reduced AGGF1 expression enhances TGF-β signaling, and induced TGF-β signaling inhibits AGGF1 expression, this further exacerbates the effect of TGF-β and aggravates arterial dilatation and remodeling. On the other hand, AGGF1 treatment reduces TGF-β signaling, and reduced TGF-β signaling increases AGGF1 expression. The feedback loop strengthens the inhibitory effect of AGGF1 on TGF-β signaling, which may explain the robust treatment effect of AGGF1 protein therapy.

We showed that inhibition of TGF-β signaling by pirfenidone treatment significantly inhibited vascular inflammation and remodeling, however, this effect was dependent on the presence of AGGF1, and lost in heterozygous *Aggf1*^+/−^-TAC mice with *Aggf1* expression reduced by only 50% (Fig. 7). This raises the hope that pirfenidone may be used to treat pressure-overload-induced vasculopathies such as thoracic aortic aneurysms and dissection in combination with AGGF1. Pirfenidone is used clinically for treatment of idiopathic pulmonary fibrosis (IPF), a rare disease with extremely poor prognosis, and reduces the risk of IPF progression or death by 35%^37^. Because vascular remodeling and other factors such as TGF-β1 reduce the expression of AGGF1 (Figs. 8b, 9d and Supplementary Figs. S1–2, S18a) and AGGF1 is required for effective therapy by pirfenidone (Fig. 7), the efficacy of pirfenidone treatment for vascular remodeling may be compromised by the reduced AGGF1 expression in diseases with reduced AGGF1 expression. Therefore, we recommend a combined treatment with both pirfenidone and AGGF1 for future therapy of IPF and vascular remodeling-induced diseases.

Many clinical trials target TGF-β1 signaling inhibition for treating cancer and fibrotic diseases, including many neutralizing antibodies, small molecule inhibitors, and antisense oligonucleotides^38^. Considering our finding that the efficacy of a TGF-β1 inhibitor therapy is dependent on the high expression level of AGGF1 (Fig. 7), it is important to measure and ensure that the expression level of AGGF1 is sufficiently high in the target tissues and cells of patients. Otherwise, the therapy may not achieve the expected therapeutic results.

One strength of the present study is that three independent mouse models, including the *Fbn1*^*C1041G/+*^ genetic model, BAPN-induced pharmacological model, and TAC model, were used to test the efficacy of AGGF1 protein therapy on TAA. Previously, doxycycline and fenofibrate were shown to inhibit abdominal aortic aneurysm (AAA) in the angiotensin II model, however, human trials showed no benefit or even faster aneurysm growth in one doxycycline trial^39^. Therefore, it may be important to test a therapeutic treatment in multiple animal models before performing human trials. It is interesting to note that AGGF1 protein therapy attenuated TAA-associated abnormalities in all three mouse models we tested. We found some differences, but also similarities, among the three animal models (Supplementary Table S3). First, *Fbn1*^*C1041G/+*^ mice and BAPN-treated mice showed more severe phenotypes than TAC mice as the dilatation of the thoracic aortas was <50% in the TAC model, but more than 50% and up to 80% in *Fbn1*^*C1041G/+*^ mice and BAPN-treated mice, respectively (Figs. 8–9 and Supplementary Figs. 19–20). Second, BAPN-treated mice were more likely to show thoracic aortic rupture, bleeding in the aortic arch, and death. Third, *Fbn1*^*C1041G/+*^ mice showed pathological thickening of the vascular media, whereas the two other models showed changes in both vascular media and adventitia. Fourth, the dilatation sites were slightly different among the three models. In TAC mice, the ascending aortas, the aortic arch and the right carotid artery were dilated. In *Fbn1*^*C1041G/+*^ mice, the dilatation occurred at the ascending aortas and the aortic arch as well as the root of ascending aortas. The dilatation sites in BAPN-treated mice included the ascending aortas and descending thoracic aortas. However, on the molecular and cellular levels, the three models shared remarkable similarities, including similar TGF-β1 signaling, vascular inflammation, and proliferation of VSMCs.

In summary, using three different mouse models for TAA, including a TAC-induced mouse model, a genetic *Fbn1*^*C1041G/+*^ TAA model, and a BAPN-induced TAA model, we demonstrated the promising therapeutic effects of intraperitoneal injection of purified AGGF1 protein (protein therapy) in attenuation of arterial dilatation and remodeling associated with TAA. We also showed that AGGF1 expression was reduced in the three mouse models and human patients affected with TAA, and that AGGF1 is critical for vascular inflammation and remodeling as haploinsufficiency of *Aggf1* or VSMC-specific KO of *Aggf1* exacerbated arterial dilatation, inflammation, and remodeling. Mechanistically, the AGGF1 receptor on VSMC, integrin α7, interacts with LAP-TGF-β1 to inhibit TGF-β1 maturation and signaling, whereas AGGF1 enhances the interaction between integrin α7 and LAP-TGF-β1, and further attenuates TGF-β1 maturation and signaling. We also show that IPF treatment agent pirfenidone attenuates TAC-induced arterial dilatation, inflammation and remodeling by inhibiting Smad3 activation and ERK1/2 activation, and this effect is dependent on AGGF1. We recommend combined pirfenidone/AGGF1 treatment as a strategy for management of IPF and other vasculopathies associated with vascular inflammation and remodeling.

## Methods

Animal care and experimental procedures were approved by the Institutional Animal Care and Use Committee at Tongji Medical College, Huazhong University of Science and Technology (No: [2014]S329). Animal experiments conformed to the guidelines of the Care and Use of Animals for Research by the Ministry of Science and Technology of the P. R. China (2006–398). Euthanasia was performed by anesthesia with injection of 3% chloral hydrate (0.1 ml/10 g body weight) followed by cervical dislocation.

The human studies were approved by the Ethics Committee of Union Hospital of Tongji Medical College at Huazhong University of Science and Technology (No: [2018]S353).

### Plasmids, siRNAs, recombinant proteins, and pirfenidone

The expression plasmid for the human *AGGF1* gene (NM_018046), pCMV-3xFLAG-AGGF1, was described previously^20,23,25,26,40^. The cDNA for *AGGF1* was amplified by PCR using plasmid pCMV-3xFLAG-AGGF1 as a template, and subcloned into the pEGFP-N1 vector, resulting in an expression plasmid for AGGF1-GFP. The deletion of the RDD motif was introduced into pCMV-3xFLAG-AGGF1 and AGGF1-GFP by PCR-based mutagenesis, resulting in expression plasmids for AGGF1-RDD^del^-FLAG and AGGF1-RDD^del^-GFP, respectively. The pET-28a-AGGF1 plasmid for expression of recombinant 6xHis-tagged AGGF1 protein was described previously^22,23,26,27^. The deletion of the RDD motif was introduced into pET-28a-AGGF1 by PCR-based mutagenesis, resulting in an expression plasmid for AGGF1-RDD^del^.

The cDNA for the human *TGFB1* gene encoding LAP-TGF-β1 (NM_000660) was amplified by PCR using mRNA and cDNA from HUVECs (human umbilical vein endothelial cells), and subcloned into the pEGFP-N1 vector or pCMV-3xFLAG-tagged vector, resulting in expression plasmids for LAP-TGF-β1-GFP and LAP-TGF-β1-FLAG. The deletion of the RGD motif was introduced into LAP-TGF-β1-GFP by PCR-based mutagenesis, resulting in expression plasmids for LAP-TGF-β1-RGD^del^-GFP.

The expression plasmids for the human *ITGA7* gene (NM_002206) encoding integrin α7 and *ITGA5* (NM_002205) encoding integrin α5 were constructed by PCR amplification of their cDNA sequences from HUVECs and subcloned into the pCMV-3xFLAG-tagged vector, resulting in expression plasmids for Integrin α7-FLAG and Integrin α5-FLAG.

All plasmids were constructed using PCR with primers and restriction sites listed in Supplementary Table S1. The PCR was performed using Phanta Max Super-Fidelity DNA Polymerase (P505-d1, Vazyme, China) according to the manufacturer’s instructions. The cloning was performed using the ClonExpress II One Step Cloning Kit (C112-01, Vazyme, China). All expression plasmids were verified by direct DNA sequencing analysis.

The sequences of siRNAs were GGAGTCTCCTGGAGATGATdTdT for *Aggf1*, CCAGACCTGTTCTACATGTdTdT for *Itga7* and TTCTCCGAACGTGTGTCACGTdTdT for negative control siRNA (siNC). The siRNAs were synthesized by RiboBio, Guangzhou, China.

Recombinant wild type 6xHis-tagged AGGF1 and mutant AGGF1-RDD^del^ were purified from *E. coli* BL21(DE3) transformed with pET-28a-AGGF1 or pET-28a-AGGF1-RDD^del^. When the transformed *E. coli* BL21(DE3) were grown to OD_600_ of 0.3-0.7, isopropyl-β-D-thiogalactoside (IPTG, 70 nM) was added to induce expression of the AGGF1 protein (22 °C, 220–250 rpm for 12 h). The bacteria are then broken under high pressure and the AGGF1 protein was purified using a Ni-NTA agarose column (Cat number L2001, Biolinkedin, China) according to the instructions from the manufacturer (BioLinkedin, China). The purified protein was examined using polyacrylamide gel electrophoresis (PAGE) and stained with Coomassie blue. If needed, the protein was further treated using Zeba Spin Desalting Columns (Cat number 89894, Thermo-Fisher Scientific, USA). The protein concentration was measured using the Protein Quantification Kit (Cat number KTD3001, Abbkine, China). TGF-β1 was purchased from PEPROTECH, America (Cat number 100-21). Pirfenidone was obtained from YuanYe, Shanghai, China (Cat number 53179-13-8).

### Cell culture and transfection

MOVAS cells (immortalized VSMCs from mouse aortas; ATCC, American Type Culture Collection) and HEK293T cells (ATCC, American Type Culture Collection) were cultured in DMEM containing 10% fetal bovine serum (FBS; Cat number #10270106, Thermo-Fisher Scientific, USA), 100 U/ml penicillin, and 100 µg/ml streptomycin. The cells were incubated at 37 °C in a humidified atmosphere of 95% O_2_ and 5% CO_2_. VSMCs were cultured into six-well plates (Cat number CCP6H, Servicebio, China), transfected with plasmid DNA or siRNA using Lipofectamine 2000 according to the manufacturer’s instructions (Cat number 11668027, Invitrogen, America), and used for Western blot analysis, quantitative real-time RT-PCR analysis or ELISA (enzyme-linked immunosorbent assays). VSMCs were also cultured and transfected in 10 cm cell culture dishes (TCD010100, Biofil, China) and used for co-immunoprecipitation assays.

HEK293T cells were cultured and transfected into 12-well plates (Cat number TCP010012, Biofil, China), and used for ELISA, or into 10 cm cell culture dishes for co-immunoprecipitation assays. Forty-eight hours after transfection, cells were harvested for Western blot, quantitative real-time RT-PCR analysis or co-immunoprecipitation assays. For ELISA, 36 hours after transfection, DMEM was replaced with a medium that did not contain 10% FBS, and cells were allowed to grow for 12 hours and then collected for the assays.

### Quantitative real-time RT-PCR analysis

The cells were transfected and grown for 48 h, washed with pre-cooled PBS for 3 times, and incubated with an appropriate amount of Trizol (Cat number 15596026, Invitrogen, USA) for 5 min. A fifth volume of chloroform was added, vortexed for 1 min, and incubated for 5 min at room temperature. The samples were centrifuged at 12,000 rpm at 4 °C for 15 minutes. The supernatant was mixed with a half volume of isopropyl alcohol, incubated for 30 min at −20 °C, centrifuged at 12,000 rpm at 4 °C for 15 min. The pellet was washed with anhydrous ethanol and 70% anhydrous ethanol, and resuspended in DEPC water. The concentration of RNA samples was measured using NANODROP 2000 (Thermo-Fisher Scientific, USA). The RNA was reverse-transcribed into cDNA using the First Strand cDNA Synthesis Kit (Cat number 11141ES10, YEASEN, China) according to the manufacturer’s instructions. Quantitative real-time PCR analysis was performed using the qPCR SYBR Green Master Mix (Low Rox Plus) (Cat number MQ10201S, Monad, China) on a QuantStudio® 12 K Flex Real-Time PCR System (Thermo, Waltham, MA, USA) according to the manufacturer’s instructions. The RNA expression level was determined using the 2^-△△CT^ method as described^41,42^. The primers for real-time RT-PCR analysis were listed in Supplementary Table S2.

### Western blot analysis

Cells or mouse tissue samples were lysed and used for Western blot analysis as described^43^. The cells were washed with pre-cooled PBS for 3 times, and then incubated with ice-cold lysis buffer (25 mM Tris-HCl (pH 8.0), 150 mM NaCl, 1% Triton X-100, 200 μM sodium deoxycholate, 1 mM dithiothreitol, 5 mM EDTA, 0.5 mM phenylmethanesulfonyl-fluoride, 10 mM N-ethylmaleimide, 10 mM iodoacetamide and a cocktail of protease inhibitors) for 20 min. The samples were centrifuged at 12,000 rpm for 10 min at 4 °C. The supernatant was mixed with loading buffer, boiled at 100 °C for 10 min, and subjected to SDS-PAGE. The mouse tissue samples were mixed with lysis buffer, and ground with glass grinding rods until the blood vessels were thoroughly ground. The samples were placed in a shaker at 4 °C, shaked at 30 rpm for 30 min, and centrifuged at 12000 rpm for 20 min at 4 °C. The supernatant was mixed with loading buffer, boiled at 100 °C for 10 min, and subjected to SDS-PAGE. The proteins were transferred to PVDF membranes. After blocking for 2 h at room temperature in TBST buffer containing 5% (w/v) BSA, the membranes were incubated under gentle agitation overnight at 4 °C with a primary antibody (dissolved in 5% (w/v) BSA according to instructions). The antibodies used include: anti-AGGF1 (Cat number 11889-1-AP, Proteintech, America), anti-β-actin (Cat number AB0035, Abway, China), anti-GAPDH (Cat number AB0038, Abway, China), anti-p-Smad3 (Cat number CY5140, Abway, China), anti-T-Smad3 (Cat number CY5013, Abway, China), anti-p-Smad2 (Cat number AP1342, ABclonal, China), anti-T-Smad2 (Cat number CY5090, Abway, China), anti-p-ERK1/2 (Cat number CY5277, Abway, China), anti-T-ERK1/2 (Cat number CY5487, Abway, China), anti-FLAG (Cat number M185-3L, MBL, Japan), anti-GFP (Cat number T0005, Affinity, America), IgG (Cat number A7028, Beyotime, China), anti-LAP-TGF-β1 (Cat number 21898-1-AP, Proteintech, America), anti-integrin α7 (Cat number A14246, ABclonal, China), and anti-mature TGF-β1 (Cat number A2124, ABclonal, China). The membranes were then washed three times for 10 min each with TBST and incubated with horseradish-peroxidase (HRP)-conjugated secondary antibodies, followed by chemiluminescence detection using ECL substrate (Cat number SQ201L, Yamei, China) according to the manufacturer’s instructions. The secondary antibodies included a goat anti-rabbit antibody (Cat number BL003A, lot number 22181594, Biosharp, China) or a goat anti-mouse antibody (Cat number BL003A, lot number 22210628, Biosharp, China). The secondary antibodies were also diluted in 5% BSA in TBST before use. Western blot band intensities were quantified by densitometry using ImageJ software.

### Co-immunoprecipitation assay (Co-IP)

Co-IP was performed as described^44–46^. Total protein extracts from transfected HEK293T cells or VSMCs were extracted with ice-cold lysis buffer (described in the Western blot analysis section above). After removing a small part of the lysed samples and adding protein loading buffer as input, the lysates were then mixed with an antibody against FLAG or GFP (1 μg/500 μl) or control IgG. After overnight incubation on a 4 °C inverted shaker, an appropriate amount of Protein A/G-PLUS-agarose (Cat number AA0142, Best Chrom, China) was added according to the Manufacturer’s instructions and incubate on a 4 °C inverted shaker for 6 h. Then, we used the lysis buffer to wash the samples 4 times with an interval of 30 min each time, and placed it on a shaker at 4 °C during the interval. The protein loading buffer was added after the last wash and boiled at 100 °C for 10 min. The samples were subjected to Western blotting analysis.

### Enzyme-linked immunosorbent assay (ELISA)

An ELISA was carried out according to the manufacturer’s instructions. HEK293T cells were cultured in 12-well plates, transfected with plasmid DNA for 24 h, and starved in the serum-free medium for 12 hours. The samples were centrifuged at 12,000 rpm for 10 min at 4 °C, and the supernatant was collected for detecting the secretion of TGF-β1 from cells using the Human TGF-β1 Detection Kit (Cat number EHC107b, NEOBIOSCIENCE, China). In experiments with AGGF1 or AGGF1-RDD^del^ treatment, HEK293T cells were cultured to 90% cell density in 12-well plates, treated with the recombinant AGGF1 protein, and starved in a serum-free medium for 12 h. The samples were centrifuged at 12,000 rpm for 10 min at 4 °C, and the supernatant was collected for ELISA.

For VSMCs, the cells were cultured in 12-well plates to 90% cell density, and the culture medium was replaced with a serum-free medium. VSMCs were then treated with a recombinant AGGF1 protein or pirfenidone for 48 h. The culture media were collected for ELISA using the Mouse TGF-β1 Detection Kit (Cat number EMC107b, NEOBIOSCIENCE, China). Similarly, for VSMCs transfected with plasmids or siRNA, 36 h after the transfection, the medium was replaced with a serum-free medium, and the transfected cells were cultured for another 12 h. The samples were centrifuged at 12,000 rpm for 10 min at 4 °C, and the supernatant was collected for ELISA using the Mouse TGF-β1 Detection Kit according to the manufacturer’s instructions.

For each ELISA experiment, we used the standard substrate in the kit to create a standard curve, and the concentration of TGF-β1 in the supernatant was calculated using the standard curve.

### Mice

Wild-type (WT) C57BL/6J mice and heterozygous *Aggf1*^*+/−*^ knockout (KO) mice with exons 2–11 deleted were described before^22,25,27,47^. *Aggf1* flox mice, *Aggf1*^*fl/fl*^mice, were generated by Cyagen (Suzhou, China). *Aggf1*^*fl/fl*^ mice were mated with Tagln-Cre mice on a C57B/L6 background (also from Cyagen) to generate vascular smooth muscle cell (VSMC)-specific KO mice for *Aggf1*, referred to as *Aggf1*^*smcKO*^. The general strategy for creation of *Aggf1*^*smcKO*^ mice is shown in Supplementary Fig. S5. *Aggf1*^*smcKO*^ mice were identified by genotyping with PCR. The primers for PCR are:

Primers for Region 1: Forward: CTCCCTTAGACAGGGTCTCAC; Reverse: CTGCAAGCTGATAATGAACTC;

Primers for Region 2: Forward: CAGGTTCTCAGTACAGAGGTAAAGGC; Reverse: GGTTGGAATAGAAGGCTTACTGAC;

Primers for the Tagln promoter: Forward: CAGACACCGAAGCTACTCTCCTTCC; Reverse: CGCATAACCAGTGAAACAGCATTGC;

Primers for the *Cre* gene: Forward: GCCTGCATTACCGGTCGATGC; Reverse: CAGGGTGTTATAAGCAATCCC.

Twelve-week-old mice were used to establish the TAC model for vascular remodeling as reported^11,19^. In brief, mice were anesthetized with 3% chloral hydrate (0.1 ml/10 g body weight) before surgery. A small incision in the mouse thoracic cavity was made to expose the aortic arch while using a small animal ventilator (ALC-V8S, ALCBIO, China) to maintain the mouse’s breathing. TAC was performed by tying a 7–0 silk suture around a 27-gauge needle overlying the arch at the point between the brachiocephalic trunk and left common carotid artery. Afterwards, the muscles and skin were sutured carefully in sequence, and the mice were allowed to wake up and used for follow-up experiments. Mice that died within the first day after surgery were excluded from further studies. In TAC models for AGGF1 or AGGF1-RDD^del^ protein therapy, we used a dose of 5 μg/10 g body weight, and administered it once every two days for 3 weeks in TAC model by intraperitoneal injection as reported^22,47^. The first injection was on the second day after surgery. Pirfenidone was dissolved in water at a concentration of 5 mg/ml, and administered orally at a dose of 0.1 ml/10 g body weight once a day as reported^48,49^. Twenty-one days after surgery, the mice were examined with echocardiography, and sacrificed. The proximal end of the right carotid artery and the ascending aortas were collected for histological analysis and Western blot analysis.

As a genetic model for TAA, *Fbn1*^*C1041G/+*^ mice with knockin (KI) of a *FBN* mutation causing Marfan syndrome were generated by Cyagen (Suzhou, China). *Fbn1*^*C1041G/+*^ mice were identified by PCR analysis and direct DNA sequencing analysis. The primers for PCR are 5′-AGCGAGCTCCCTTAACAGTG-3′ (forward) and 5′-ATTGTTAGCCCCTCAGTGCC-3′ (reverse). The forward primer was also used for direct DNA sequencing analysis. *Fbn1*^*C1041G/+*^ mice at the age of 10 weeks were treated with an initial intraperitoneal injection of the purified AGGF1 protein (5 μg/10 g body weight) followed by intraperitoneal injection of AGGF1 once every two days for 8 weeks. At 18 weeks of age, the mice were examined with echocardiography, and sacrificed. The blood vessels from the ascending aortas to the aortic arch were collected for histological analysis and Western blot analysis.

To develop a pharmacological model for TAA, three-week-old C57B/L6 mice were fed with a normal diet and 0.25% β-aminopropionitrile (BAPN) (Cat number A9090, Aladdin, China) dissolved in water for 4 weeks as reported. The mice were treated with an initial intraperitoneal injection of the purified AGGF1 protein (5 μg/10 g body weight) when BAPN was started, followed by intraperitoneal injection of AGGF1 once every two days for 4 weeks. The mice were examined with echocardiography, and sacrificed. The blood vessels from the ascending aortas to the descending branch of the thoracic aortas were collected for histological analysis and Western blot analysis.

All mice were maintained in IVCs at the density of 3–5 mice per cage in an SPF animal room with a temperature of 22–25 °C, a humidity of 50–60%, and a dark/light cycle of 12 h. Cages were cleaned once a week, and the feeding formula is listed in Supplemental Table S5. For development of TAC models and BAPN models for TAA, male mice and female mice were studied separately, and age- and weight-matched mice in each group were randomized into the sham mice and TAC mice, and the water control mice and BAPN mice, respectively, before TAC surgery or BAPN treatment. For all AGGF1 treatment studies, the mice were matched by age, weight and sex and randomized into different treatment groups, and male mice and female mice were studied separately. Sample size determination was performed before each study using nQuery (*n* = 7 mice per group are needed to observe a significant difference for two groups with means different by twofold).

The number of mice used are listed for individual Figures as follows:

(1) For experiments with wild type and mutant *Fbn1*^*C1041G/+*^ mice, the survival rate was 100% during the entire study period and the numbers are indicated in the related Fig. 8 and Supplementary Fig. S19.

(2) For experiments with BAPN-treated mice, the survival rate was shown in Fig. 9 and Supplementary Fig. S20.

(3) For TAC mice, the mice were randomized into sham groups and TAC groups, and the surgical procedures were then performed for all mice. The numbers of mice used in studies were as follows:

In Fig. 1, 12 mice/group were prepared for 2 sham groups and 20 mice/group for 2 TAC groups. After surgery, the survival rate of mice in two sham groups was 100%, while the survival rate of mice in the TAC groups was 75% for the WT-TAC group and 75% for the KO-TAC group.

In Fig. 2, 12 mice/group were prepared for 2 sham groups and 20 mice/group for 2 TAC groups. After surgery, one mouse in the CTL-sham group died due to the procedure, and the survival rate was 91.6%; the survival rate of SmcKO-Sham mice was 100%. After surgery, the survival rate of CTL-TAC and SmcKO-TAC groups was both 55%.

In Figs. 3, 10 mice/group were prepared for 3 sham groups and 20 mice/group for 3 TAC groups. The survival rate of the three sham groups after surgery was 100%. After surgery, the survival rate of TAC groups was 50% for the buffer group, 65% for the AGGF1 group and 60% for the AGGF1-RDD^del^ group.

In Fig. 7, 20 mice/group were prepared for 4 TAC groups. The survival rate of all groups of mice after surgery was 60%.

In Supplementary Fig. S3, 10 mice/group were prepared for 4 sham groups and 20 mice/group were prepared for 4 TAC groups. After surgery, the survival rate of mice in the sham groups was 100%, while that of mice in TAC groups was 50%, 50%, 55% and 55%, respectively.

In Supplementary Fig. S6, 12 mice/group were prepared in 2 sham groups and 20 mice/group for 2 TAC groups. After surgery, one mouse in the CTL-sham group died, and the survival rate was 91.6%; the survival rate of SmcKO-Sham mice was 100%. After surgery, the survival rate of CTL-TAC and SmcKO-TAC groups was 70% and 65%, respectively.

In Supplementary Fig. S9, 12 mice/group were prepared for 3 sham groups and 20 mice/group for 3 TAC groups. After surgery, two mice in sham groups died, and the survival rate was 91.6%, 91.6% and 100%, respectively. After surgery, the survival rate of TAC groups was 60%, 55%, and 60%, respectively.

In Supplementary Fig. S11, 10 mice/group were prepared for 3 sham groups and 20 mice/group for 3 TAC groups. The survival rate of the sham groups after surgery was 100%. After surgery, the survival rate of TAC groups was 50% in the buffer group, 65% in the AGGF1 group and 60% in the AGGF1-RDDdel group.

In Supplementary Fig. S13, 12 mice/group were prepared for 3 sham groups and 20 mice/group for 3 TAC groups. After surgery, two mice in sham groups died, and the survival rate was 91.6%, 91.6% and 100%, respectively. After surgery, the survival rate of TAC groups was 60%, 55% and 60%, respectively.

### Echocardiography

Echocardiography was performed with a Vevo 2100 High Resolution Micro Ultrasound System (Visual Sonics, Toronto, Canada) by a researcher who was blinded to treatments while mice were anesthetized with 3% chloral hydrate (0.1 ml/10 g body weight) as reported by us^19,47,50^. Briefly, after anesthesia of a mouse, the chest and neck were treated with depilatory agents, and images of the ascending aortas and carotid artery (including left and right carotid artery) were acquired on the long axis by using the MS400 MicroScan sensor on the Vevo 2100 system. The flow velocity of the left and right carotid arteries was recorded. After the image signal was stabilized, at least 3–5 cycles of peak systolic velocity were recorded and averaged for statistical analysis. In TAC models, the diameter of the ascending aortas (halfway from where the sinotubular junction to the brachiocephalic artery takes off) and carotid artery (end of the common carotid artery) was measured during end diastole. In *Fbn1*^*C1041G/+*^ mice, the diameter of the ascending aortas (halfway from where the sinotubular junction to the brachiocephalic artery takes off) was measured during end diastole. In BAPN model, the diameter of the ascending aortas near the brachiocephalic artery was measured during end diastole. An average of three measurements were performed from each animal. Echocardiography was performed by a researcher who was blinded to the animal’s genotypes and treatment status.

### Histology and immunohistochemistry

Carotid arteries and ascending aortas were excised, fixed with 4% paraformaldehyde overnight, paraffin-embedded, and sectioned (3 mm). Cross-sections of arteries were stained by haematoxylin and eosin (H&E), Sirius red or antibodies (immunostaining) as described^22,25,27,47,51,52^. For immunostaining, the sections were deparaffinized, and antigen was retrieved. 3% hydrogen peroxide was used to block peroxidase. After washing with PBS buffer for three times, the sections were blocked with 3% BSA for 30 minutes. The primary antibody was added and incubated with sections overnight at 4 °C on a shaker, followed by addition and incubation with the secondary antibody at room temperature for 60 minutes. After washing with PBS buffer for three times, the DAB chromogenic solution (Cat number G1212, Servicebio, China) was added, and the nuclei were counterstained with hematoxylin. The slides were dehydrated, mounted, and photographed under a florescence microscope or a confocal microscope. The primary antibodies used in this study include anti-AGGF1 (Cat number 11889-1-AP, Proteintech, America), anti-α-SMA (Cat number GB111364, Servicebio, China), anti-MCP1 (Cat number GB11199, Servicebio, China), and anti-CD68 (Cat number GB11067, Servicebio, China).

### Human subjects

Thoracic aortic tissue samples were collected from TAA patients undergoing surgical resection of the aortic wall at Wuhan Union Hospital Department of Cardiovascular Surgery. TAA patients had a maximum thoracic aortic diameter of between 5.0 and 6.5 cm. The non-TAA thoracic aortic tissue samples were obtained from healthy donors undergoing ascending aortic trimming before heart transplantation. There was no significant difference for the age, sex, medications and other characteristics between the two groups. The patients themselves or their families provided informed consent for donation. This study protocol was approved by the Ethics Committee of Wuhan Union hospital of Tongji Medical College, Huazhong University of Science and Technology (No: [2018]S353) and performed in compliance with the Declaration of Helsinki. Written informed consent from obtained from the study subjects. All information of TAA subjects or non-TAA subjects was provided in Supplementary Table S4.

### Statistical analysis

Quantitative data were shown as mean ± SEM. The difference between two groups of variables was compared by the unpaired two-tailed Student’s *t*-test and comparison of multiple groups (≥3 groups) was performed by one- or two-way ANOVA with Tukey’s multiple-comparison tests. A log-rank test was used to compare Kaplan–Meier survival curves between two groups of mice. A *P* value of <0.05 was considered to be statistically significant.

### Reporting summary

Further information on research design is available in the Nature Portfolio Reporting Summary linked to this article.

## Supplementary information

Supplementary Information

Reporting Summary

## Data Availability

The data that support the conclusions of this study are provided in the article or the online supplementary data, and also available from the corresponding authors. Source data are provided with this paper.

## Source data

Source Data
