# Supplementary material for: AGGF1 therapy inhibits thoracic aortic aneurysms by enhancing integrin α7-mediated inhibition of TGF-β1 maturation and ERK1/2 signaling
Source: Nat Commun. 2023 Apr 20;14:2265. doi: 10.1038/s41467-023-37809-x (PMC10119315; doi:10.1038/s41467-023-37809-x)
Supplement: Supplementary file 1 — Supplementary Information [file 41467_2023_37809_MOESM1_ESM.pdf]

Supplementary Figures

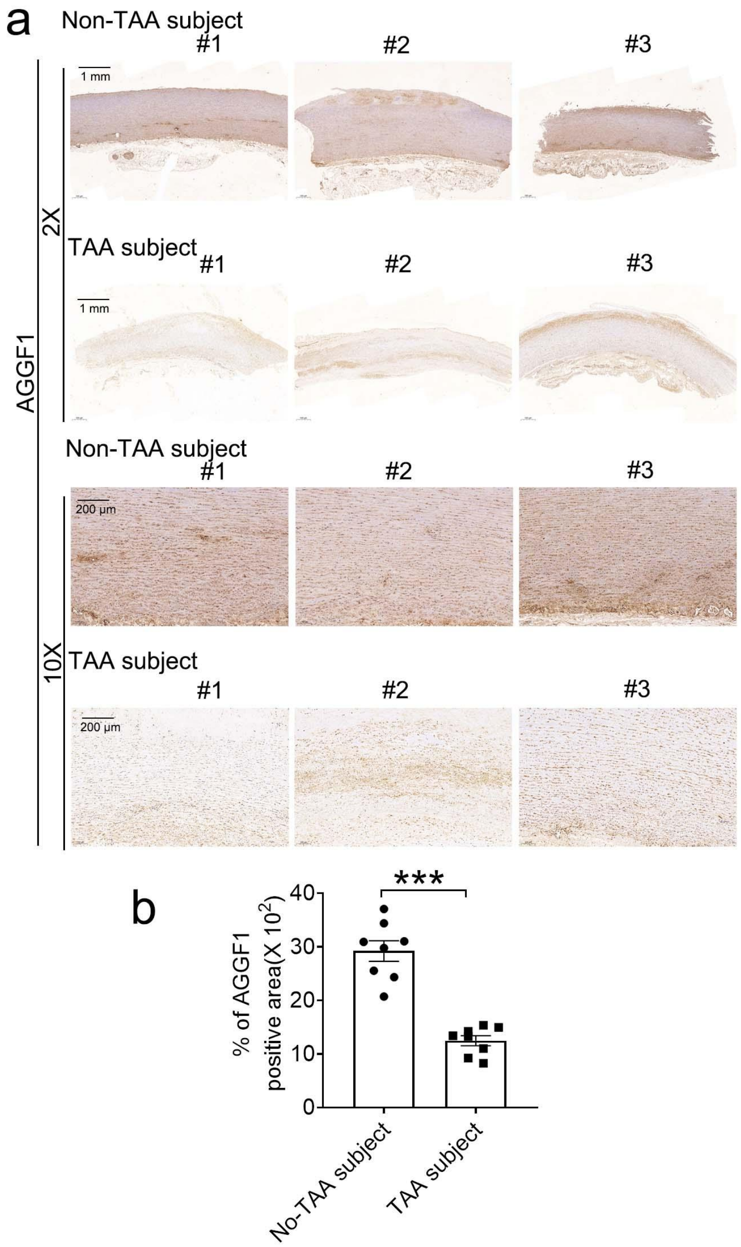

Supplementary Figure S1. The AGGF1 level is reduced in the lesion area of ascending aortas from patients with thoracic aortic aneurysms (TAA).

a. Representative immunostaining images for AGGF1 with cross-sections of the ascending aortas from patients with TAA and non-TAA control individuals. Images at 2X magnification are shown at the top and those at 10X magnification are shown at the bottom. **b.** Quantification of AGGF1 immunostaining signal intensity from images as in (**a**). *, P<0.05; **, P<0.01; ***, P<0.001; n=8 patients/group. Quantitative data were shown as mean ± SEM. Statistical analysis was performed with two-tailed unpaired Student's t-tests (**b**).

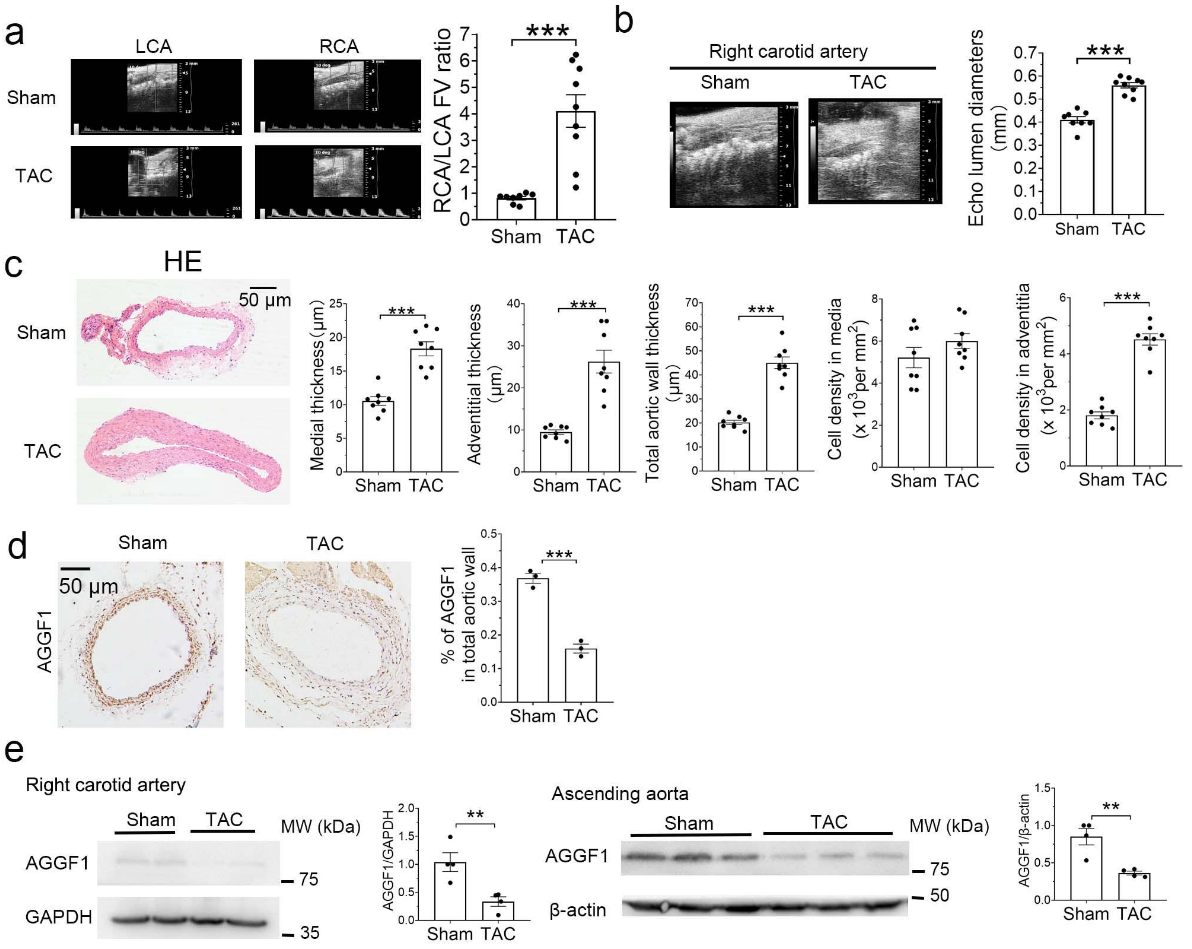

Supplementary Figure S2. TAC induces arterial dilatation and remodeling of the right carotid artery and decreases expression of AGGF1 in male mice.

a. Echocardiographic images of the left and right carotid arteries from TAC mice (LCA vs. RCA). The bar graph on the right shows the ratio of blood flow velocity (FV) of RCA over LCA three weeks after TAC. RCA, right carotid artery; LCA, left carotid artery; Sham, sham-operated mice; TAC, transverse aortic constriction (n=8 mice/group). **b.** Mean lumen diameter of RCA three weeks after TAC or Sham. Representative images are shown on the left and quantitative data are graphed on the right (n=8 mice/group). **c.** H&E staining of cross-sections of RCA three weeks after TAC or Sham (magnification, 200x). Bar graphs on the right show total aortic wall thickness (1st graph), medial layer thickness (3nd graph), adventitial layer thickness (2rd graph), cell density in media (4th graph), and cell density in adventitia (5th graph) (n=8 mice/group). **d.** Immunostaining for AGGF1 in cross-sections of RCA three weeks after TAC or Sham. Quantitative data are graphed on the right (n=3 experiments/group). **e.** Representative Western blotting images for AGGF1 with RCA and ascending aorta three weeks after TAC or Sham (n=4 experiments/group). *, P<0.05; **, P<0.01; ***, P<0.001; Quantitative data were shown as mean ± SEM. Statistical analysis was were performed with two-tailed unpaired Student's t-tests **(a-g)**.

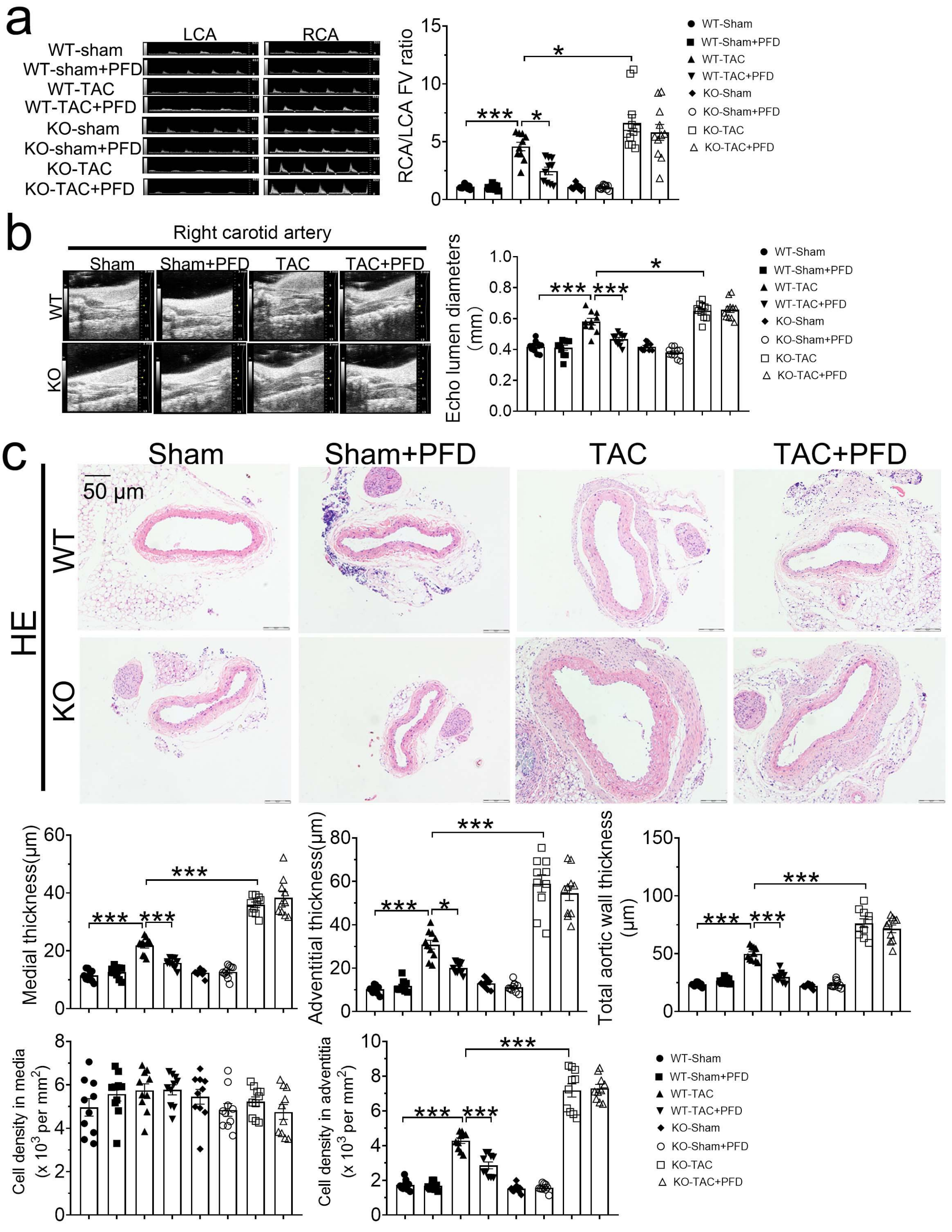

Supplementary Figure S3. *Aggf1* haploinsufficiency aggravates TAC-induced arterial dilatation and remodeling, and pirfenidone (PFD) attenuates vasculopathy in female *Aggf1*$^{+/+}$ (WT) mice, but not in female *Aggf1*$^{+/-}$ (KO) mice.

a. Representative echocardiographic images of LCA and RCA from *Aggf1*$^{+/+}$ (WT) or *Aggf1*$^{+/-}$ (KO) mice three weeks after TAC or sham operation with or without pirfenidone treatment. The bar graph on the right shows the ratio of flow velocity (FV) of RCA over LCA (n= 10, 10, 10, 10, 10, 10, 11, and 11 mice, respectively). **b.** Mean lumen diameter of RCA from WT or KO mice (n= 10, 10, 10, 10, 10, 10, 11, and 11 mice, respectively). **c.** H&E staining of cross-sections of RCA. Bar graphs

show medial thickness, adventitial thickness, total aortic wall thickness, cell density in media, and cell density in adventitia (n=10 mice/group). *, *P*<0.05; **, *P*<0.01; ***, *P*<0.001; Quantitative data were shown as mean ± SEM. Statistical analysis was performed with two-way ANOVA with Tukey's tests (**a-c**).

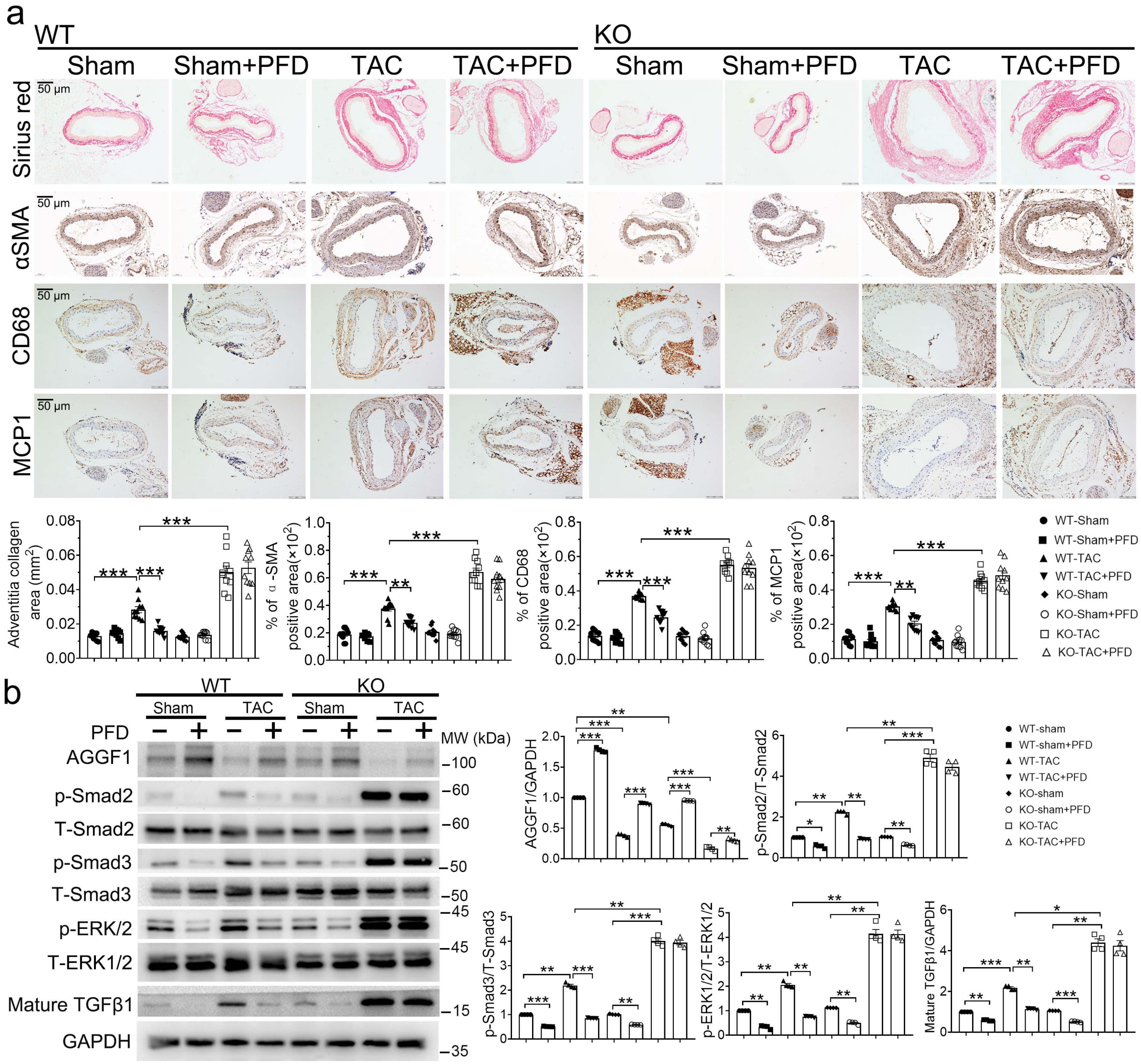

Supplementary Figure S4. *Aggf1* haploinsufficiency aggravates TAC-induced vascular remodeling, inflammation, and TGFβ1 signaling, and pirfenidone (PFD) attenuates vasculopathy in female *Aggf1*$^{+/+}$ (WT) mice, but not in female *Aggf1*$^{+/-}$ (KO) mice.

a. Sirius red staining and immunostaining of α-smooth muscle actin (α-SMA) for smooth muscle cells, inflammation marker MCP-1 and macrophage marker CD68 with cross-sections of RCA. The bar graphs at the bottom show quantification of the collagen area, and the percentage of α-SMA-positive areas, MCP1 positive cell areas, and CD68 positive cell areas. **b.** Representative Western blotting images for AGGF1, p-ERK1/2, T-ERK1/2, p-Smad2, T-Smad2, p-Smad3, T-Smad3, and mature TGF-β1. GAPDH was used as loading control. Quantified data are graphed on the right. *, *P*<0.05; **, *P*<0.01; ***, *P*<0.001; n=10 mice/group for mouse studies and n=4/group for Western blot analysis. Quantitative data were shown as mean ± SEM. Statistical analysis was performed with two-way ANOVA with Tukey's tests (**a, b**).

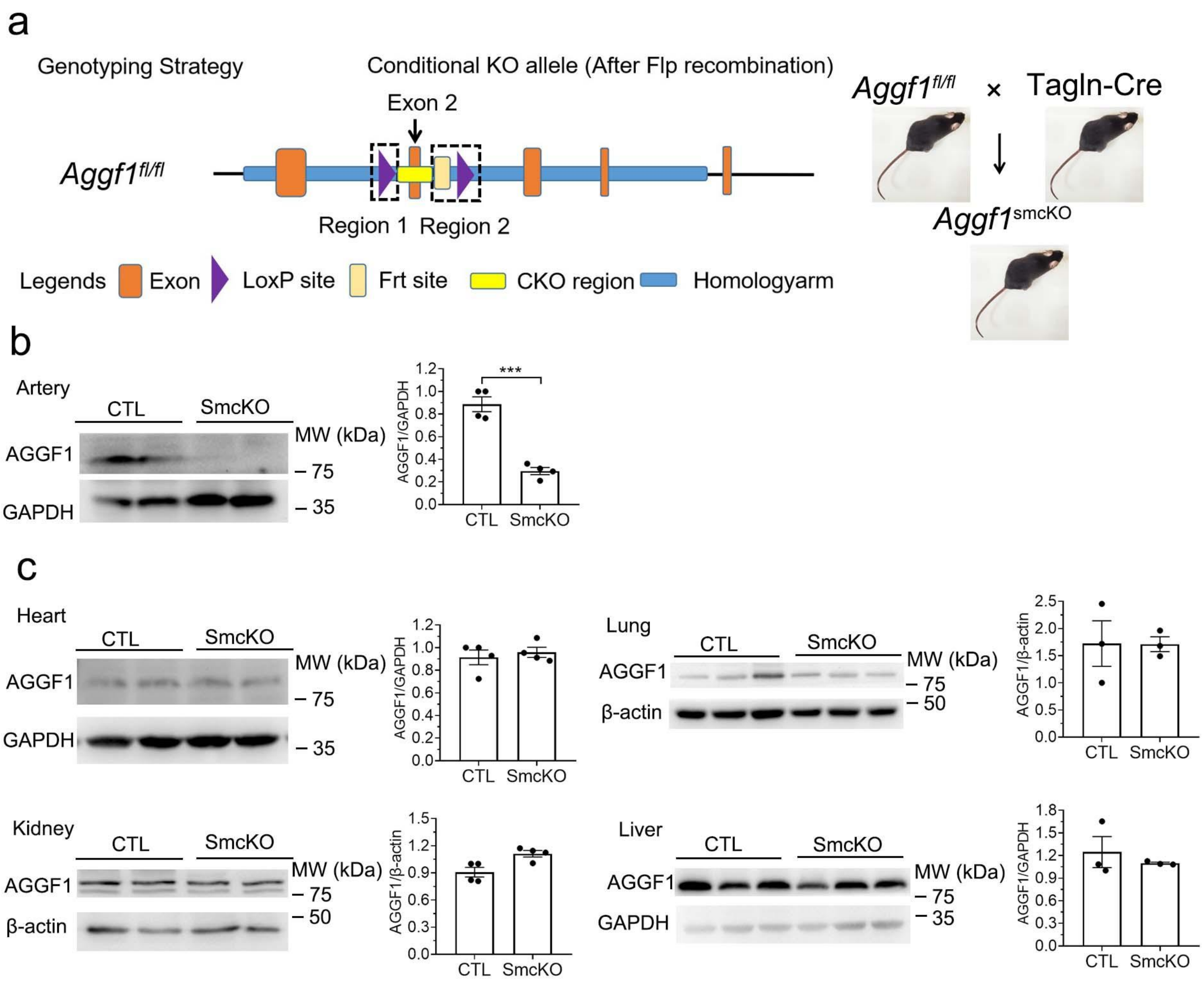

Supplementary Figure S5. Development of vascular smooth muscle cell specific knockout mice for *Aggf1*.

a. General strategy for creation of *Aggf1* flox mice (*Aggf1$^{fl/fl}$*). *Aggf1$^{fl/fl}$* mice were bred with *Tagln-Cre* mice to generate *Aggf1^{smcKO}* mice (smooth muscle specific knockout mice for *Aggf1*). Two loxP sites are located on either side of exon 2 of the *Aggf1* gene. Exon 2 can be removed when the *Tagln* promoter drives the expression of the Cre-recombinase. **b.** Western blot analysis for AGGF1 using arteries from *Aggf1$^{fl/fl}$* mice and *Aggf1^{smcKO}* mice. GAPDH was used as loading control. AGGF1 expression was markedly decreased in arteries from *Aggf1^{smcKO}* mice. **c.** Western blot analysis for AGGF1 using the heart, lung, kidney, and liver samples from *Aggf1$^{fl/fl}$* mice and *Aggf1^{smcKO}* mice. No significant difference was detected for AGGF1 expression in the heart, lung, kidney, and liver samples between *Aggf1$^{fl/fl}$* mice and *Aggf1^{smcKO}* mice. ***, $P<0.001$; n=3 experiments/group for lung and liver tissue studies and n=4/group for other studies. Quantitative data were shown as mean ± SEM. Statistical analysis was performed with two-tailed unpaired Student's t-test (**b, c**).

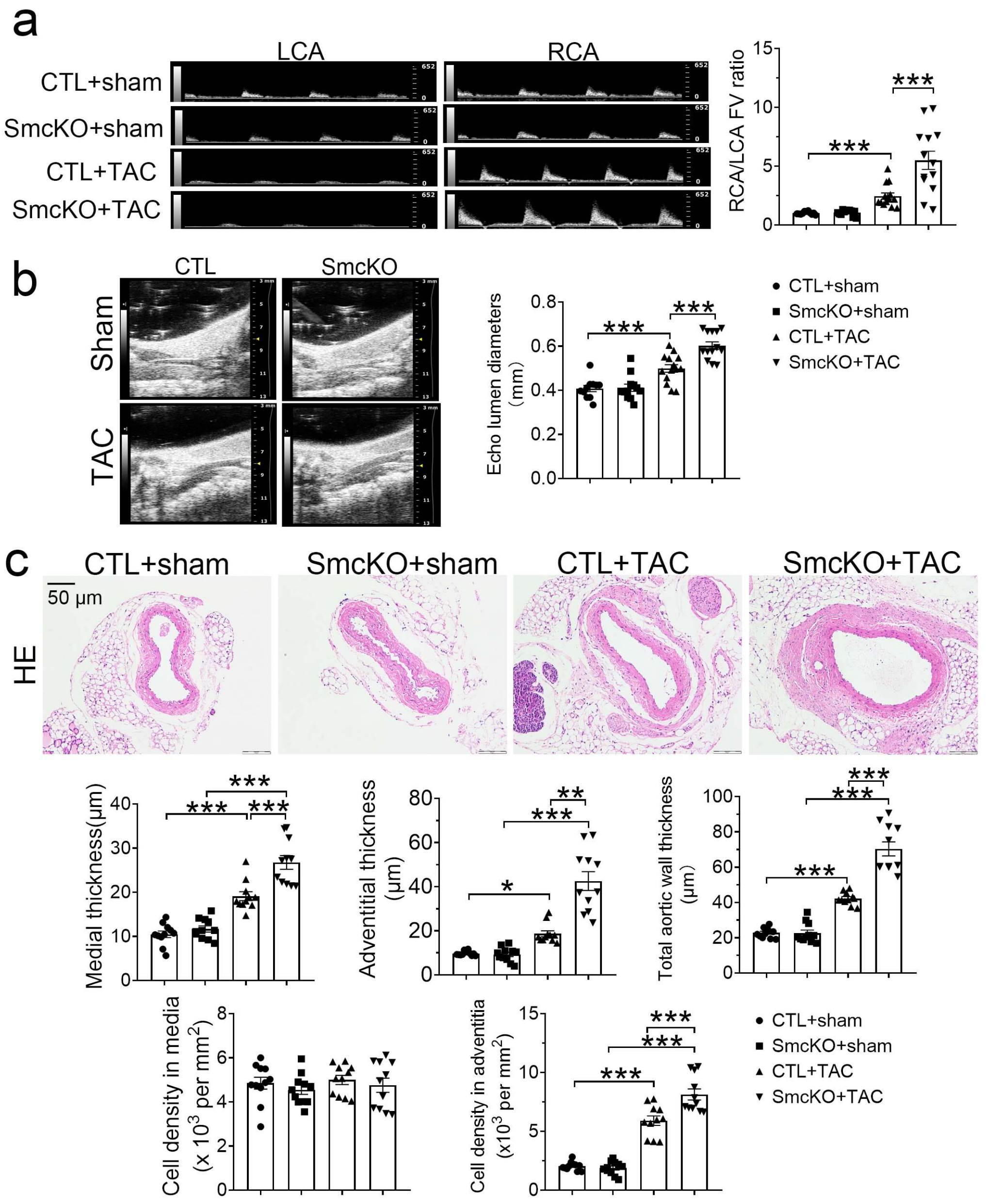

Supplementary Figure S6. VSMC-specific KO of *Aggf1* aggravates TAC-induced arterial dilatation and remodeling in female mice.

a. Echocardiographic images of LCA and RCA from *Aggf1*$^{fl/fl}$ and *Aggf1*^{smcKO} female mice three weeks after TAC or sham operation. The bar graph on the right shows the ratio of flow velocity (FV) of RCA over LCA (n= 11, 12, 14, and 13 mice, respectively). b. Mean lumen diameter of RCA (n= 11, 12, 14, and 13 mice, respectively). c. H&E staining of cross-sections of RCA, and quantification data on medial thickness, adventitial thickness, total aortic wall thickness, cell density in media, and cell density in adventitia (n=11 mice/group). *, $P<0.05$; **, $P<0.01$; ***, $P<0.001$; Quantitative data were shown as mean ± SEM. Statistical analysis was performed with two-way ANOVA with Tukey's tests (a-c).

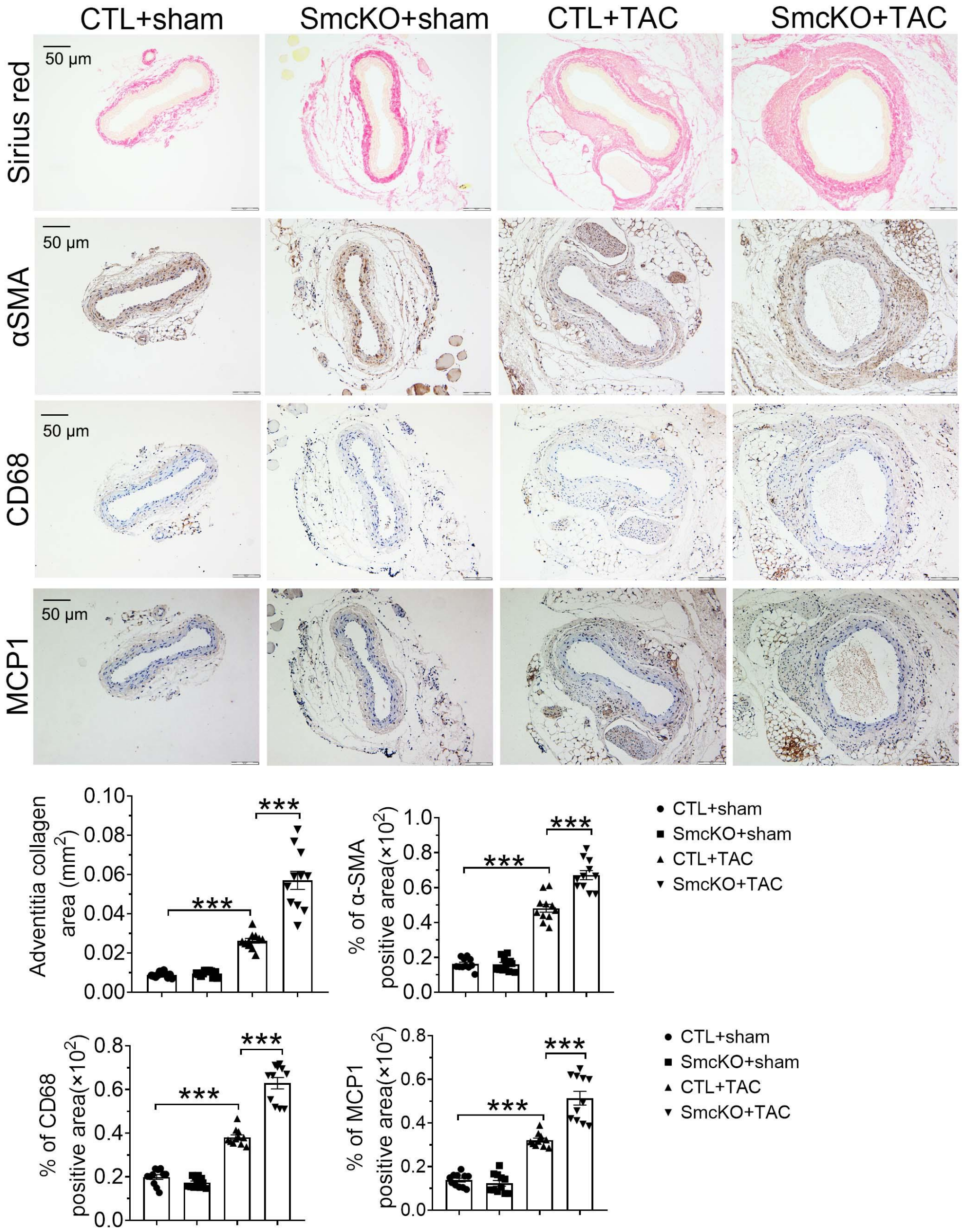

Supplementary Figure S7. VSMC-specific KO of *Aggf1* aggravates TAC-induced vascular remodeling and inflammation in female mice.

Top: Sirius red staining and immunostaining for α-SMA, MCP-1 and CD68 with cross-sections of RCA. Bottom: The bar graphs show quantification of the collagen area, and the percentage of α-SMA/CD68/MCP1 positive areas. *, P<0.05; **, P<0.01; ***, P<0.001; n=11 mice/group. Quantitative data were shown as mean ± SEM. Statistical analysis was performed with two-way ANOVA with Tukey's tests.

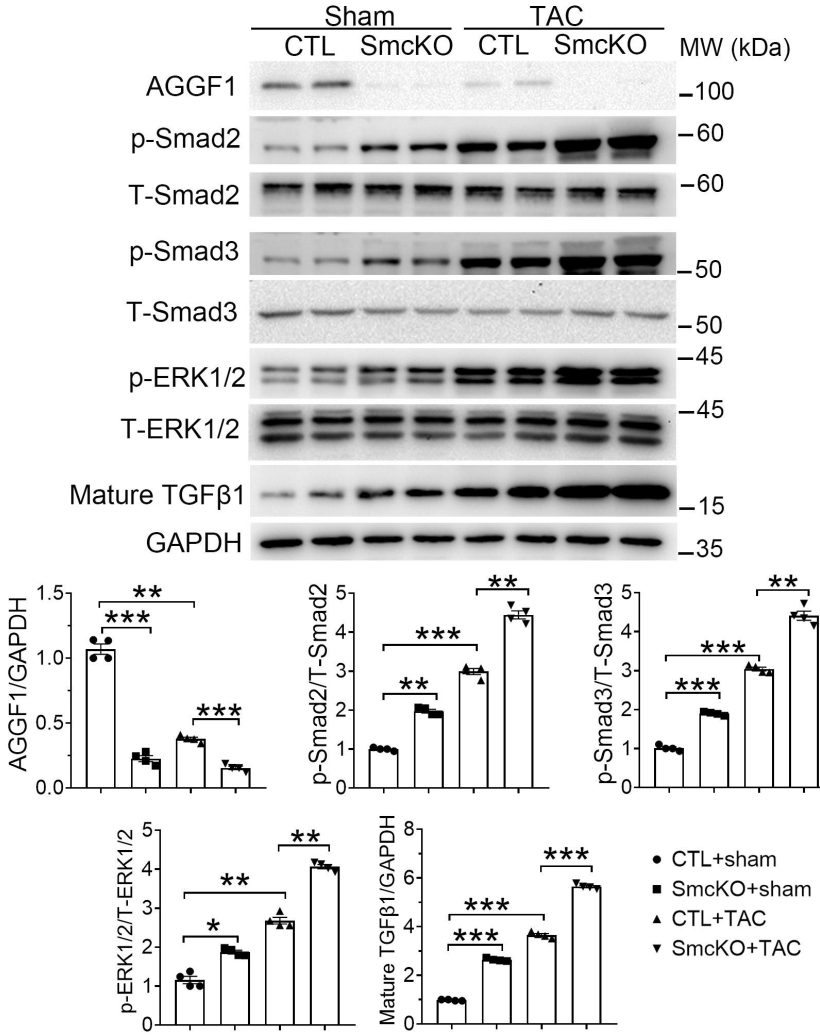

Supplementary Figure S8. VSMC-specific KO of *Aggf1* aggravates TAC-induced TGF-β1 signaling in female mice.

Western blot analysis was performed for AGGF1, p-ERK1/2, T-ERK1/2, p-Smad2, T-Smad2, p-Smad3, T-Smad3, and mature TGF-β1 using RCA samples. GAPDH was used as loading control. Quantified data are graphed at the bottom. *, $P<0.05$; **, $P<0.01$; ***, $P<0.001$; n=4 experiments/group. Quantitative data were shown as mean ± SEM. Statistical analysis was performed with two-way ANOVA with Tukey's tests.

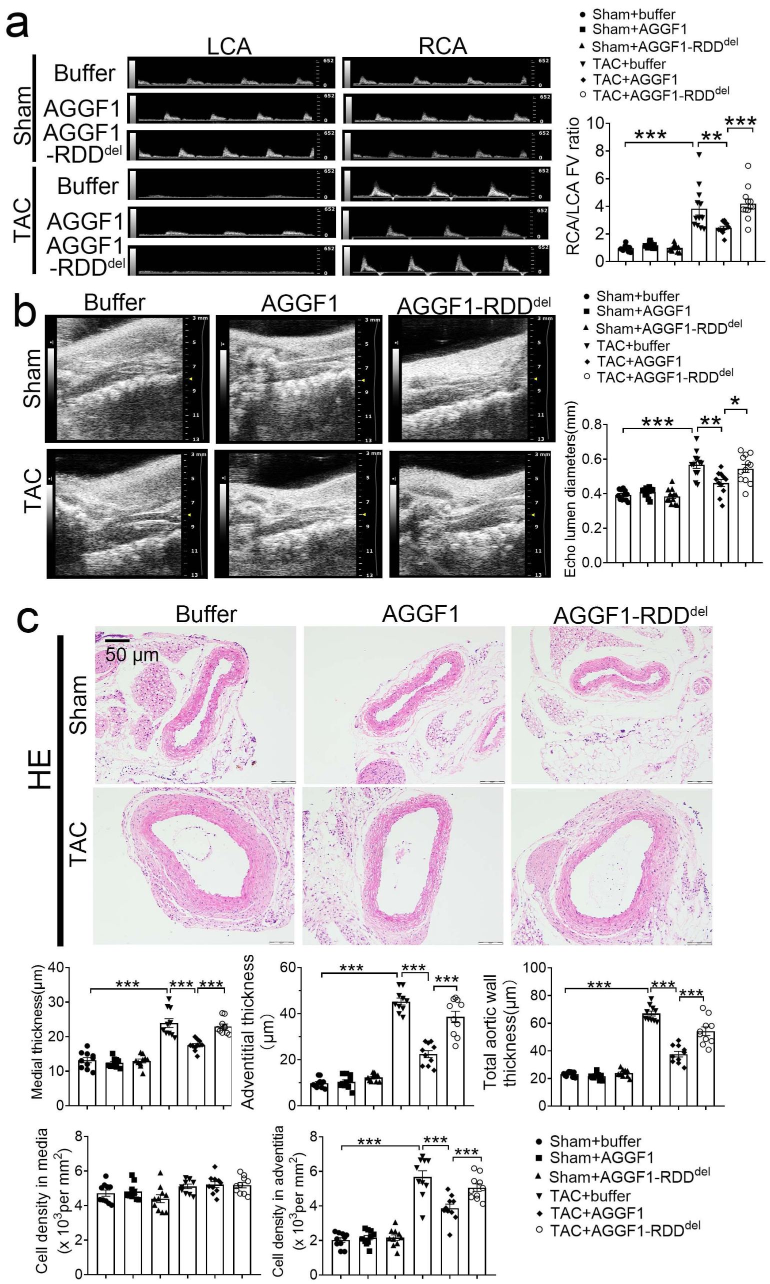

Supplementary Figure S9. Intraperitoneal injection of the purified human AGGF1 protein attenuates TAC-induced arterial dilatation and remodeling in female mice.

a. Representative echocardiographic images of LCA and RCA from mice three weeks after TAC or sham operation and treated with or without wild type AGGF1 or mutant AGGF1-RDD^{del}. The bar graph on the right shows the ratio of flow velocity (FV) of RCA over LCA (n=1, 11, 12, 12, 11, and 12 mice, respectively). **b.** Mean lumen diameter of RCA (n= 11, 11, 12, 12, 11, and 12 mice, respectively). **c.** H&E staining of cross-sections of RCA. Bar graphs at the bottom show medial thickness, adventitial thickness, total aortic wall thickness, cell density in media, and cell density in adventitia (n=10 mice/group). *, *P*<0.05; **, *P*<0.01; ***, *P*<0.001; Quantitative data were shown as mean ± SEM. Statistical analysis

was performed with two-way ANOVA with Tukey's tests (**a-c**).

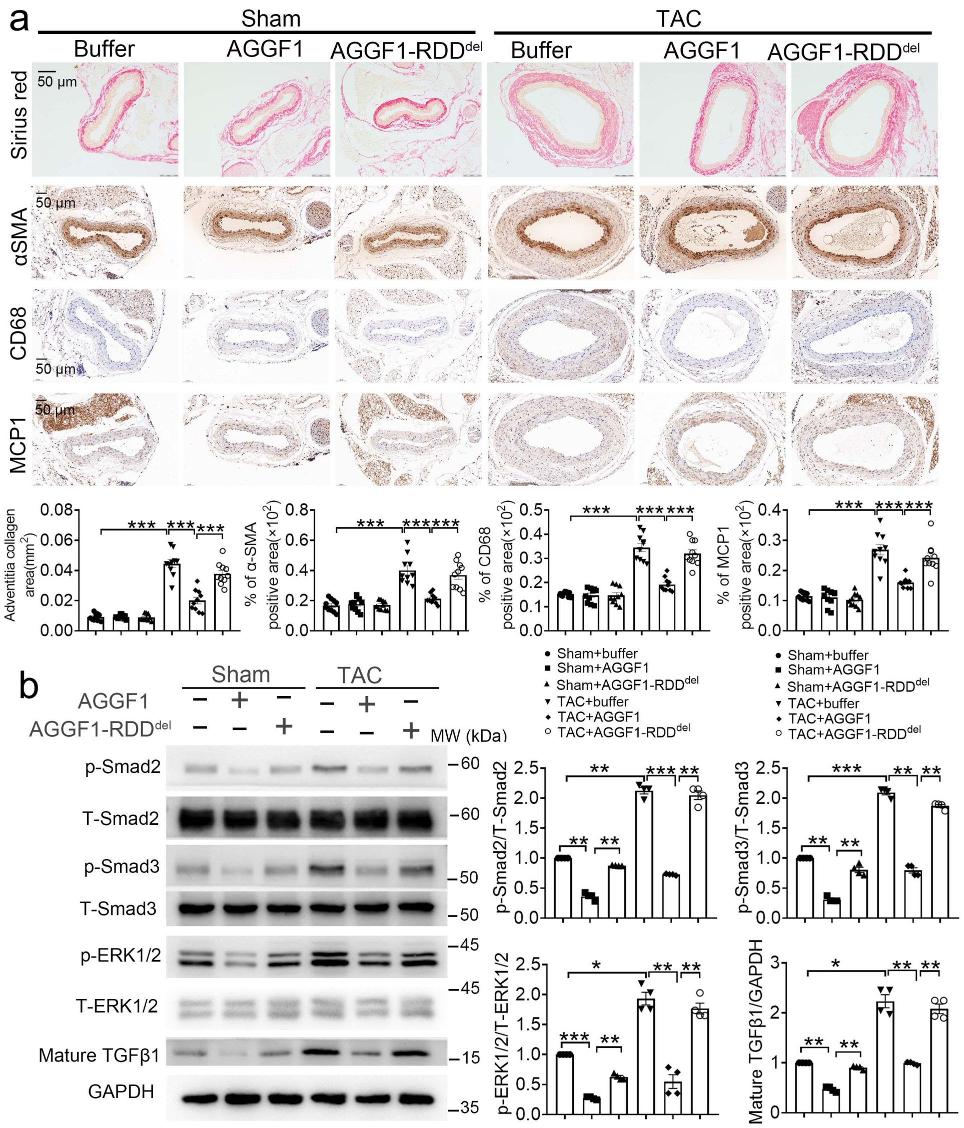

Supplementary Figure S10. Intraperitoneal injection of the purified human AGGF1 protein attenuates TAC-induced vascular inflammation, TGF-β1 maturation and signaling, and ERK1/2 signaling in female mice.

a. Sirius red staining and immunostaining for α-SMA, MCP-1 and CD68 with cross-sections of RCA. The bar graphs at the bottom show quantification of the collagen area, and the percentage of α-SMA/CD68/MCP1 positive areas. **b.** Western blot analysis for p-ERK1/2, T-ERK1/2, p-Smad2, T-Smad2, p-Smad3, T-Smad3, and mature TGF-β1 of RCA. GAPDH was used as loading control. Quantified data are graphed on at the bottom. *, P<0.05; **, P<0.01; ***, P<0.001; n=10 mice/group for mouse studies and n=4 experiments/group for Western blot analysis. Quantitative data were shown as mean ± SEM. Statistical analysis was performed with two-way ANOVA with Tukey's tests (**a, b**).

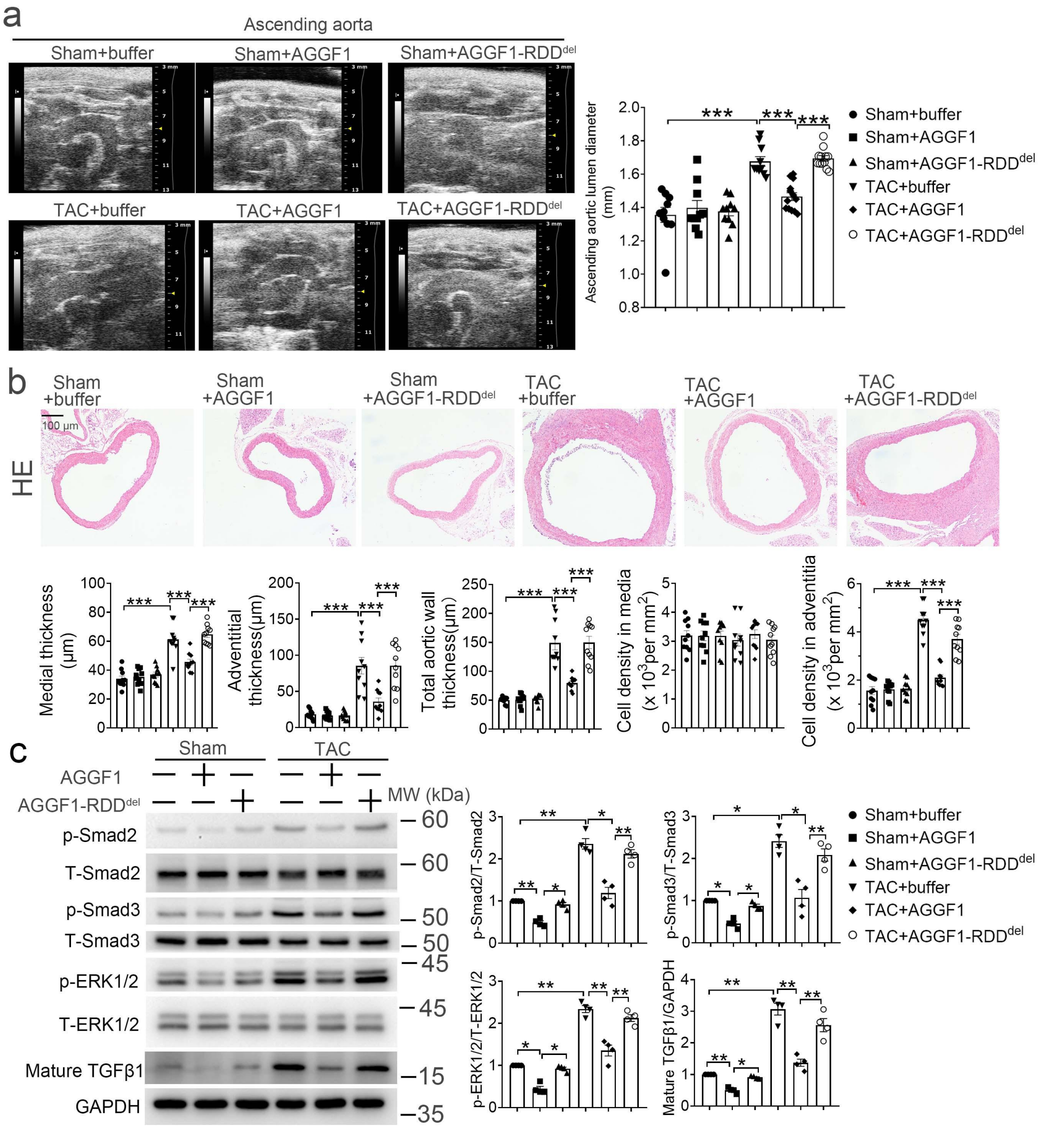

Supplementary Figure S11. Intraperitoneal injection of the purified AGGF1 protein attenuates TAC-induced arterial dilatation and remodeling, TGF-β1 signaling and ERK1/2 signaling in the ascending aortas in an RDD-dependent manner in male mice.

a. Mean lumen diameter of the ascending aortas from mice underwent TAC or Sham operation for three weeks and treated with or without wild type AGGF1 or mutant AGGF1-RDD^{del} without the RDD motif. Representative images are shown on the left and quantitative data are graphed on the right (n= 10, 10, 10, 10, 13, and 12 mice, respectively). **b.** H&E staining of cross-sections of the ascending aortas from mice underwent TAC or Sham operation for three weeks and treated with or without wild type AGGF1 or mutant AGGF1-RDD^{del}. Bar graphs at the bottom show medial thickness (1nd graph), adventitial thickness (2st graph), total aortic wall thickness (3rd graph), cell density in media (4th graph), and cell density in adventitia (5th graph) (n=10 mice/group). **c.** Western blot analysis for p-ERK1/2, T-ERK1/2, p-Smad2, T-Smad2, p-Smad3, T-Smad3, and mature TGF-β1 in the ascending aortas. GAPDH was used as loading control. Quantified data are graphed on the

right (n=4 experiments/group). *, $P<0.05$; **, $P<0.01$; ***, $P<0.001$; Quantitative data were shown as mean ± SEM. Statistical analysis was performed with two-way ANOVA with Tukey's tests (**a-c**).

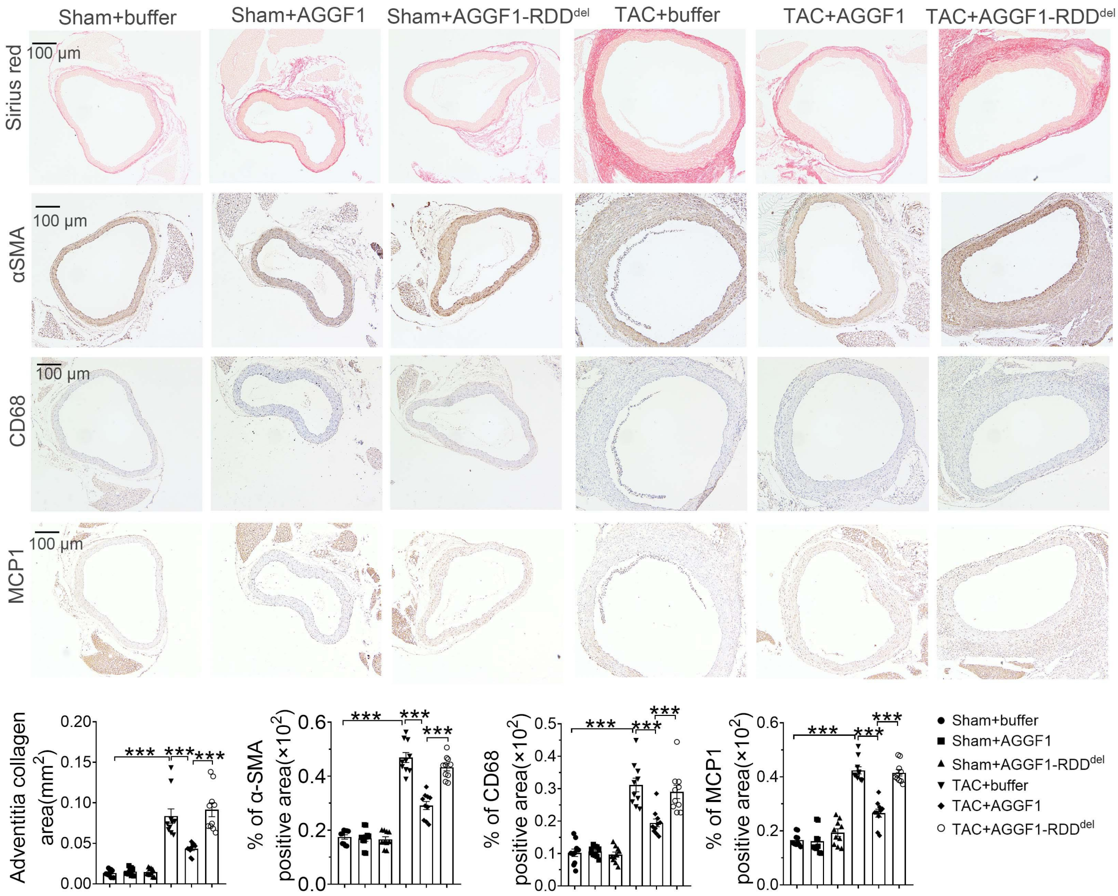

Supplementary Figure S12. Intraperitoneal injection of the purified AGGF1 protein attenuates TAC-induced arterial remodeling and inflammation of the ascending aortas in an RDD-dependent manner in male mice. Representative Sirius red staining of cross-sections of the ascending aorta, and immunostaining for α-SMA, inflammation marker MCP-1 and macrophage marker CD68 are shown at the top. Bar graphs for the percentage of the collagen areas, and percentage of α-SMA-, CD68-, and MCP1-positive areas are shown at the bottom. *, $P<0.05$; **, $P<0.01$; ***, $P<0.001$; n=10 mice/group. Quantitative data were shown as mean ± SEM. Statistical analysis was performed with two-way ANOVA with Tukey's tests.

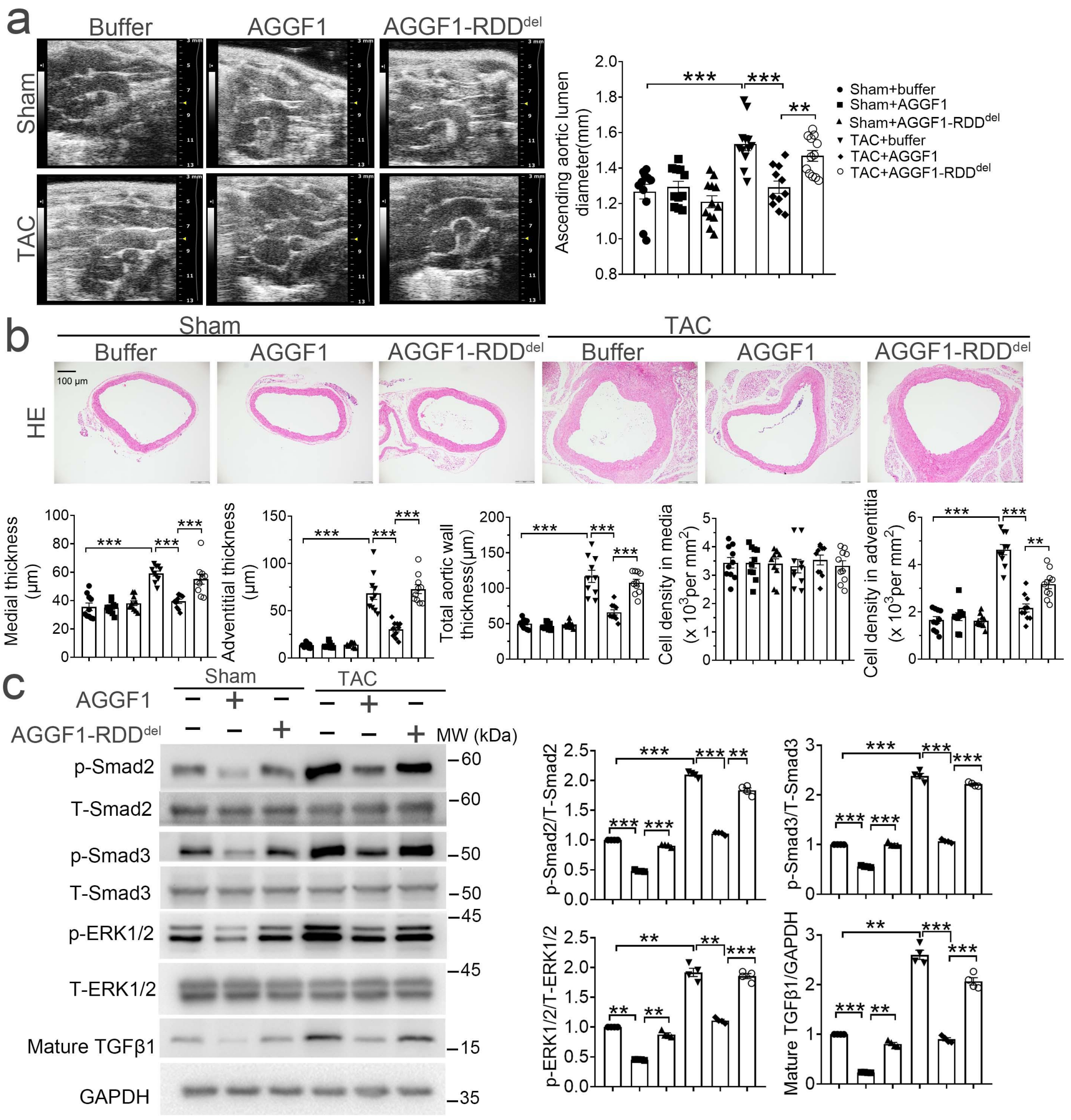

Supplementary Figure S13. Intraperitoneal injection of the purified AGGF1 protein attenuates TAC-induced arterial dilatation and remodeling, TGF-β1 signaling and ERK1/2 signaling of the ascending aortas in an RDD-dependent manner in female mice. **a.** Mean lumen diameter of the ascending aortas from mice underwent TAC or Sham operation for three weeks and treated with or without wild type AGGF1 or mutant AGGF1-RDD^{del} without the RDD motif. Representative echocardiographic images are shown on the left and quantitative data are shown on the right (n= 11, 11, 12, 12, 11, and 12 mice, respectively). **b.** H&E staining of cross-sections of the ascending aortas from mice underwent TAC or Sham operation for three weeks and treated with or without wild type AGGF1 or mutant AGGF1-RDD^{del}. Bar graphs at the bottom show medial thickness, adventitial thickness, total aortic wall thickness, cell density in media, and cell density in adventitia (n=10 mice/group). **c.** Western blot analysis for p-ERK1/2, T-ERK1/2, p-Smad2, T-Smad2, p-Smad3, T-Smad3, and mature TGF-β1 of ascending aortas. GAPDH was used as loading control. Quantified data are graphed on the right (n=4 experiments/group). *, *P*<0.05; **,

P<0.01; ***, *P*<0.001; Quantitative data were shown as mean ± SEM. Statistical analysis was performed with two-way ANOVA with Tukey's tests (**a-c**).

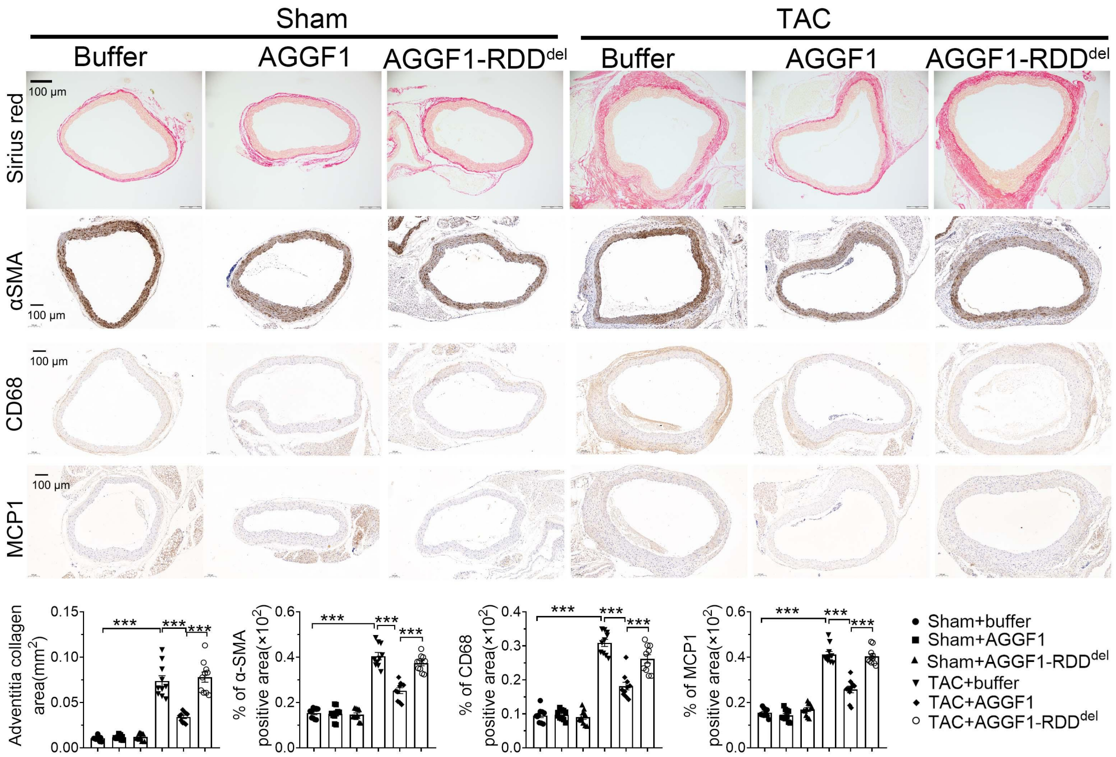

Supplementary Figure S14. Intraperitoneal injection of the purified AGGF1 protein attenuates TAC-induced arterial remodeling and inflammation of the ascending aortas in an RDD-dependent manner in female mice.

Representative Sirius red staining images of cross-sections of the ascending aortas, and immunostaining images for α-SMA， inflammation marker MCP-1 and macrophage marker CD68 are shown at the top. Bar graphs for the percentage of the collagen areas, and percentage of α-SMA-, CD68-, and MCP1-positive areas are shown at the bottom. *, *P*<0.05; **, *P*<0.01; ***, *P*<0.001; n=10 mice/group. Quantitative data were shown as mean ± SEM. Statistical analysis was performed with two-way ANOVA with Tukey's tests.

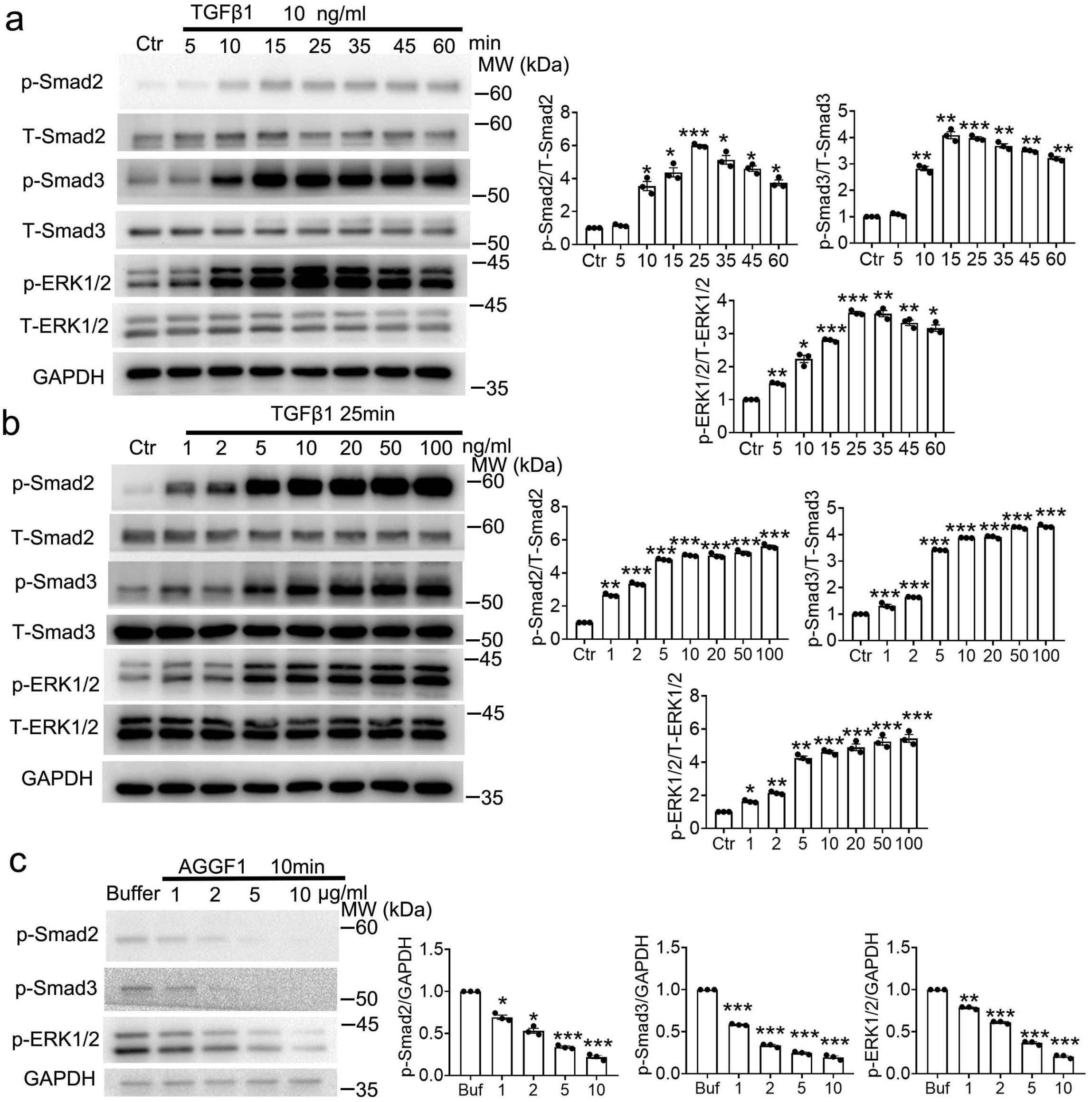

Supplementary Figure S15. AGGF1 inhibits TGF-β1 and ERK1/2 signaling in concentration-dependent manners in VSMCs.

a. Western blot analysis for p-ERK1/2, T-ERK1/2, p-Smad2, T-Smad2, p-Smad3, and T-Smad3 in VSMCs treated with TGF-β1 (10 ng/ml) for different time duration. **b.** Western blot analysis for p-ERK1/2, T-ERK1/2, p-Smad2, T-Smad2, p-Smad3, and T-Smad3 in VSMCs treated with different concentrations of TGF-β1 for 25 minutes. **c.** Western blot analysis for p-ERK1/2, p-Smad2, and p-Smad3 in VSMCs treated with different concentrations of AGGF1 for 10 min. GAPDH was used as a loading control. Quantified data are graphed on the right. Ctr, control; Buf, elution buffer for AGGF1 purification. *, $P<0.05$; **, $P<0.01$; ***, $P<0.001$; n=3 experiments/group. Quantitative data were shown as mean ± SEM. Statistical analysis was performed with one-way ANOVA with Tukey's tests (**a-c**).

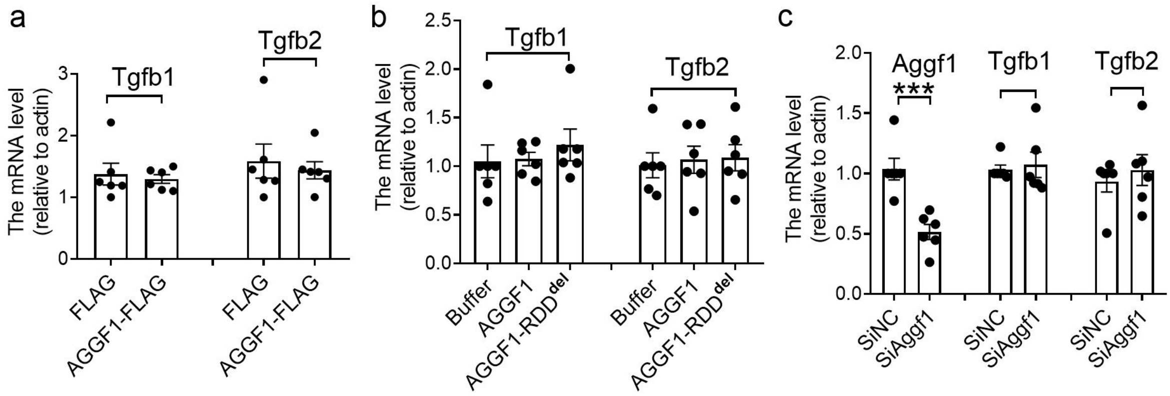

Supplementary Figure S16. AGGF1 does not affect the mRNA expression of *Tgfb1* and *Tgfb2*. **a.** Real time RT-PCR analysis for measuring the mRNA levels of *Tgfb1* and *Tgfb2* in VSMCs transfected with an expression plasmid for AGGF1-FLAG or control empty vector (FLAG). **b.** The mRNA levels of *Tgfb1* and *Tgfb2* in VSMCs treated with wild type AGGF1 or mutant AGGF1-RDD^{del}. Elution buffer (Buffer) was used as the negative control. **c.** The mRNA levels of *Tgfb1* and *Tgfb2* in VSMCs transfected with *Aggf1* siRNA or control siNC. Gene expression levels were normalized to *β-actin*. *, P<0.05; **, P<0.01; ***, P<0.001; n=6 experiments/group. Quantitative data were shown as mean ± SEM. Statistical analysis was performed with two-way ANOVA with Tukey's tests (**b**) or two-tailed unpaired Student's t-tests (**a, c**).

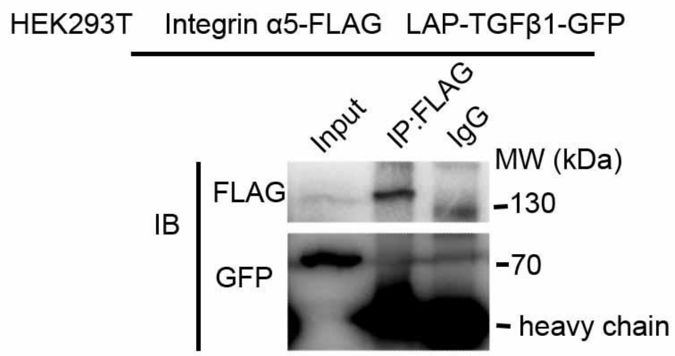

Supplementary Figure S17. Co-immunoprecipitation analysis for integrin α5 and TGF-β1. Co-IP was carried out using HEK293T cells with overexpression of TGF-β1-GFP and integrin α5-FLAG. An antibody recognizing integrin α5-FLAG failed to precipitate TGF-β1-GFP (The experiment was replicated three times).

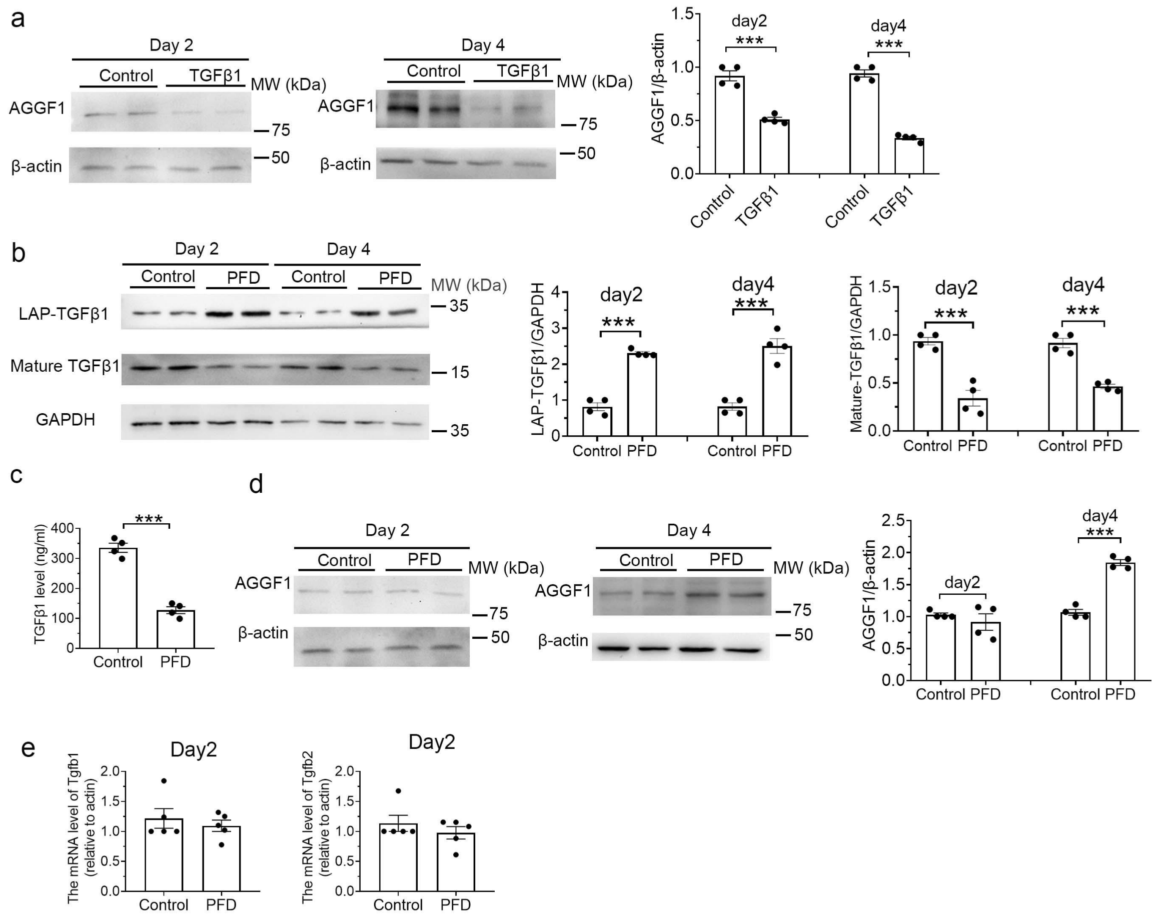

Supplementary Figure S18. TGF-β1 inhibits AGGF1 expression and pirfenidone (PFD) increases AGGF1 expression.

a. Western blot analysis for AGGF1 in VSMCs treated with TGF-β1 for 2 days and 4 days. VSMCs were starved for 12 hours and then stimulated with TGF-β1. **b.** Western blot analysis for LAP-TGF-β1 and mature TGF-β1 in VSMCs treated with PFD for 2 days and 4 days. **c.** ELISA for measurement of TGF-β1 in culture media from VSMCs treated with PFD for 48 hours. **d.** Western blot analysis for AGGF1 in VSMCs treated with PFD for 2 days and 4 days. Quantitative data for Western blotting are graphed on the right. **e.** Real time RT-PCR analysis for measuring the mRNA levels of *Tgfb1* and *Tgfb2* in VSMCs treated with PFD for 48 hours. Gene expression levels were normalized to *β-actin*. *, $P<0.05$; **, $P<0.01$; ***, $P<0.001$; n=5 experiments/group for Real time RT-PCR analysis and n=4 experiments/group for other studies. Quantitative data were shown as mean±SEM. Statistical analysis was performed with two-tailed unpaired Student's t-tests (**a-e**).

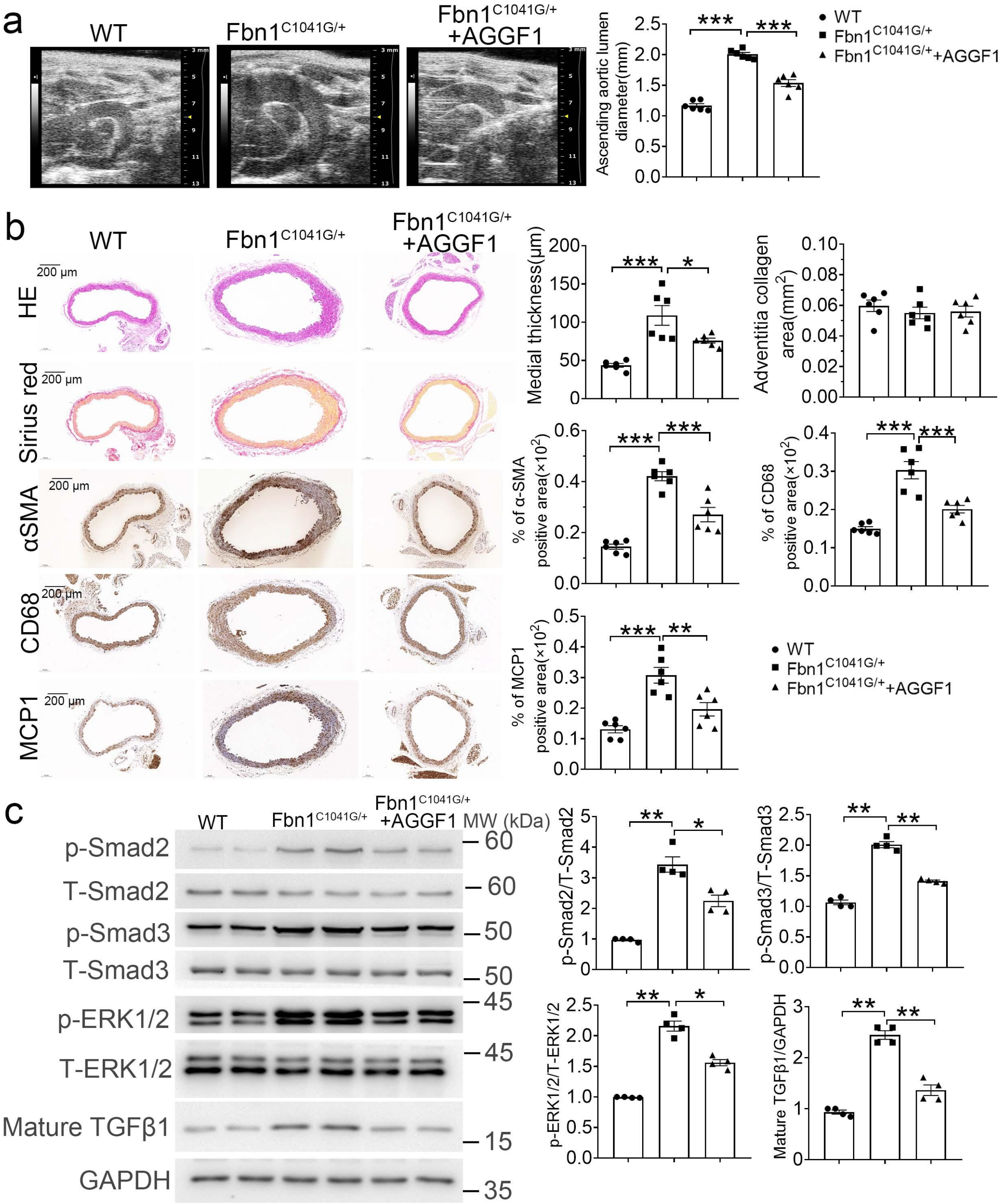

Supplementary Figure S19. AGGF1 attenuates arterial remodeling, inflammation and TGF-β1 signaling in a genetic model for TAA (female *Fbn1*$^{C1041G/+}$ mice).

a. Mean lumen diameter of the ascending aortas from three groups of mice, including wild type mice (WT), heterozygous *Fbn1*$^{C1041G/+}$ mice treated with PBS buffer, and *Fbn1*$^{C1041G/+}$ mice treated with recombinant AGGF1 protein. **b.** H&E staining, Sirius red staining and immunostaining for α-SMA, MCP-1 and CD68 with cross-sections of ascending aortas. Bar graphs at the right show quantification of medial thickness, the collagen area, and the percentage of α-SMA/CD68/MCP1 positive areas. **c.** Western blot analysis for p-ERK1/2, T-ERK1/2, p-Smad2，T-Smad2，p-Smad3，

T-Smad3 and mature TGF-β1. GAPDH was used as loading control. *, $P<0.05$; **, $P<0.01$; ***, $P<0.001$; n=6 mice/group for mouse studies and n=4 experiments/group for Western blot analysis. Quantitative data were shown as mean ± SEM. Statistical analysis was performed with two-way ANOVA with Tukey's tests (**a-c**).

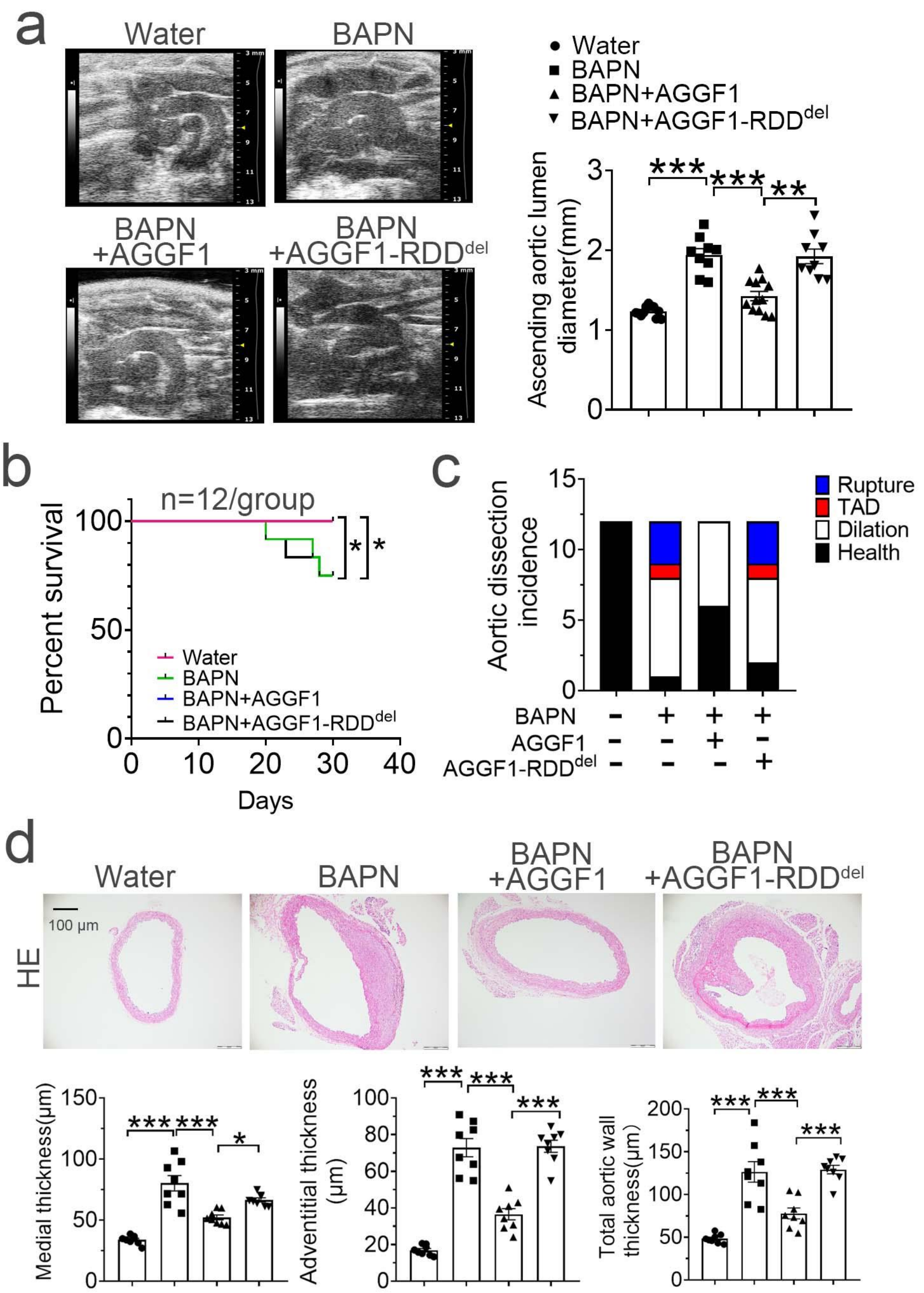

Supplementary Figure S20. AGGF1 blocks arterial remodeling in a BAPN-induced model for aortic aneurysms in female mice.

a. Mean lumen diameter of the ascending aortas from four groups of male mice (control mice fed with water, BAPN-fed mice treated with PBS buffer, BAPN-fed mice treated with wild type AGGF1, and BAPN-fed mice treated with mutant AGGF1 with a deletion of the RDD motif (n= 12, 9, 12, and 9 mice, respectively). **b.** Kaplan-Meier survival curves and statistical analysis using a log-rank test. **c** Ratios of mice with thoracic aortic dissection (TAD), thoracic aortic rupture, and thoracic aortic dilatation among four different groups of mice. **d** H&E staining and Sirius red

staining of cross-sections of ascending aortas. Bar graphs at the bottom show quantification of medial thickness, adventitial thickness, and total aortic wall thickness (n=8 mice/group). *, $P<0.05$; **, $P<0.01$; ***, $P<0.001$; n≥9 mice/group. Quantitative data were shown as mean ± SEM. Statistical analysis was performed with two-way ANOVA with Tukey's tests (**a, d**).

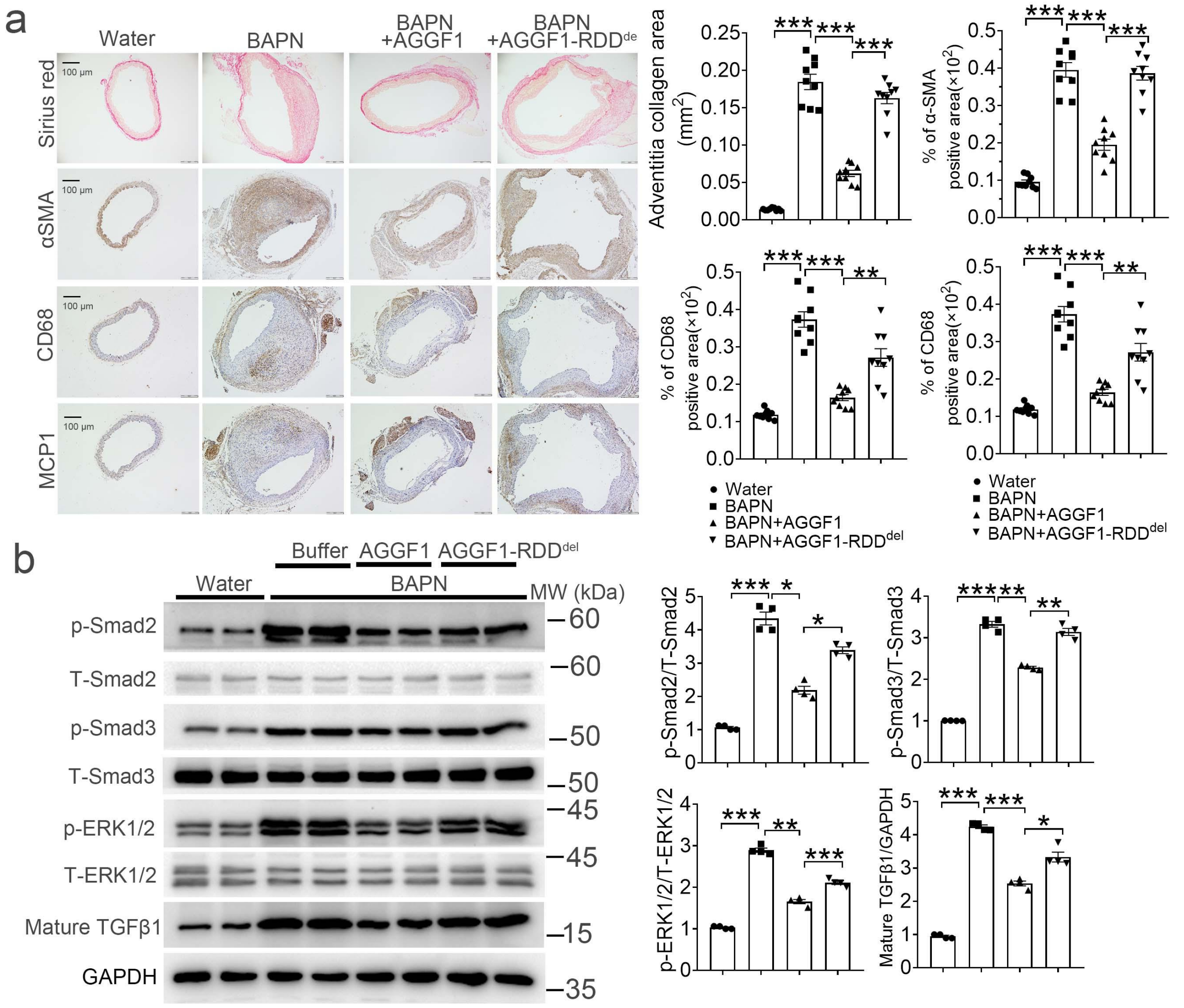

Supplementary Figure S21. AGGF1 blocks arterial remodeling, inflammation and TGF-β1 signaling in a BAPN-induced model for aortic aneurysms in female mice.

a. Representative Sirius red staining and immunostaining images for α-SMA, MCP-1 and CD68 with cross-sections of ascending aortas. Bar graphs on the right show quantification of the collagen area, and the percentage of α-SMA/CD68/MCP1 positive areas. **b.** Western blot analysis for p-ERK1/2, T-ERK1/2, p-Smad2, T-Smad2, p-Smad3, T-Smad3 and mature TGF-β1. GAPDH was used as loading control. *, $P<0.05$; **, $P<0.01$; ***, $P<0.001$; n=9 mice/group for mouse studies and n=4 experiments/group for Western blot analysis. Quantitative data were shown as mean±SEM. Statistical analysis was performed with two-way ANOVA with Tukey's tests (**a, b**).

Supplementary Table S1. Sequences of primers and restriction digestion sites for construction of different plasmids

Plasmid	Primer	Restriction site	Sequence (5'-3')
TGFβ1-FLAG	Forward	Hind III	aaggatgacgatgacaagcttatgccgccctccgggct
	Reverse	Kpn I	tcctctagagtcgactggtaccgctgcacttgcaggagcgca
TGFβ1-GFP	Forward	Xho I	agcgctaccggactcagatctcgagatgccgccctccgggct
	Reverse	Hind III	cgactgcagaattcgaagcttgctgcacttgcaggagcgcac
AGGF1-GFP	Forward	Xho I	agcgctaccggactcagatctcgagatggcctcggaggcgcc
	Reverse	Hind III	cgactgcagaattcgaagctttcactctaaagtcccttttacccaagg
AGGF1-RDD^{del}-FLAG	Forward	Hind III	tgaaggaactttccaagctcctgcatctgttc
	Reverse	Kpn I	tgaacagatgcaggagcttggaaagttccttca
AGGF1-RDD^{del}-GFP	Forward	Xho I	tgaaggaactttccaagctcctgcatctgttc
	Reverse	Hind III	tgaacagatgcaggagcttggaaagttccttca
Integrin α7-FLAG	Forward	Hind III	aaggatgacgatgacaagcttatggccggggctcggagc
	Reverse	Kpn I	tcctctagagtcgactggtaccctaggcggtgcctggccctg
Integrin α5-FLAG	Forward	Hind III	aaggatgacgatgacaagcttatggggagccggacgcca
	Reverse	Kpn I	cctctagagtcgactggtacctcaggcatcagaggtggctg

Supplementary Table S2. Primers for real-time RT-PCR analysis

Gene	Primer	Sequence (5’-3’)
Mouse *Actb*	Forward	cgtccacccgcgagcacag
	Reverse	cgacgaccagcgcagcgatat
Mouse *Aggf1*	Forward	aacgcagggagcaggtgggaag
	Reverse	ccccgtttccaccccatcttctc
Mouse *Il6*	Forward	ctgcaagagacttccatccag
	Reverse	agtggtatagacaggtctgttgg
Mouse *Mcp1*	Forward	ttgtcaccaagctcaagagaga
	Reverse	gaggtggttgtggaaaaggtag
Mouse *Mmp2*	Forward	ttccccccgcaagcccaagtg
	Reverse	gcccgagcaaaagcatcatccac
Mouse *Mmp9*	Forward	cgtgccggaagcgctcatgtac
	Reverse	gtgggaggtgctgtcggctgtg
Mouse *Itga7*	Forward	caggggagctaacgtgcagaaggag
	Reverse	gccaatcacatcccgagtctccaa
Mouse *Tgfb*1	Forward	cccgcgtgctaatggtggacc
	Reverse	agcaatgggggttcgggcact
Mouse *Tgfb*2	Forward	cgagcggagcgacgaggagtact
	Reverse	tggggttttgcaagcggaagac

Supplementary Table S3. Comparison of three mouse models for TAA.

	**TAC**	***Fbn1***^{C1041G} **mice**	**BAPN-induced TAA**
**Dilatation**	Less than 1.5 times	More than 1.5 times	More than 1.5 times
**Rupture and dissection**	No	No	Common
**Vascular thickening**	Media and adventitia	Media	Media and adventitia
**Signaling pathway**	TGF-β1	TGF-β1	TGF-β1
**Pathogenesis**	Hypertension	Fibrillin 1 mutation	Lysyloxidase inhibition
**Inflammation**	Yes	Yes	Yes
**VSMCs proliferation**	Yes	Yes	Yes
**Location**	From the ascending aorta to the right carotid artery	From the root of the aorta to the aortic arch	From ascending aorta to descending thoracic aorta

Supplementary Table S4. Characteristics of non-TAA subjects and TAA patients.

Characteristics	Non-TAA subjects (n=8)	TAA subjects (n=8)	*P* value
Age (year)	45.7±3.9	48.2±7.8	0.7786
Number of Males	5 (62.5%)	6 (75%)	0.9999
Presentation			
Hypertension	3 (37.5%)	4 (50%)	0.9999
Diabetes	1 (12.5%)	1 (12.5%)	0.9999
Stroke	3 (37.5%)	2 (25%)	0.9999
Coronary heart disease	1 (12.5%)	2 (25%)	0.9999
Hyperlipidemia	1 (12.5%)	3 (37.5%)	0.5692
Smoking	2 (25%)	2 (25%)	0.9999
Medication			
Antiplatelet agents	2 (25%)	2 (25%)	0.9999
Anticoagulants	0 (0%)	0 (0%)	0.9999
ACE inhibitor or ARB	3 (37.5%)	3 (37.5%)	0.9999
Beta-blockers	1 (12.5%)	2 (25%)	0.9999
Calcium-channel blockers	2 (25%)	3 (37.5%)	0.9999
Statins	1 (12.5%)	2 (25%)	0.9999

Data for the age are shown as mean ± SEM, and other data are shown as the number of subjects. TAA, thoracic aortic aneurysm tissue; Non-TAA, age-matched study subjects undergoing heart transplantation surgery without TAA; ACE, angiotensin-converting enzyme; ARB, angiotensin receptor blockers. Comparisons were performed with two-tailed unpaired Student’s t-tests (Age) or Fisher's exact tests (other information).

Supplementary Table S5. Formula for reproductive feeding and maintenance feeding of mice (WQJX BIO-TECHNOLOGY, Wuhan, China).

Breeding feeding

Corn : 35.5%	Calcium hydrophosphate: 2%
Bean pulp: 23%	Mountain flour:1.5%
Imported fish meal: 8%	Composite mineral salt:0.7%
Flour:20%	Decavitamin:0.3%
Yeast:8%	Prepowder:1%

Maintenance feeding

Corn : 40%	Calcium hydrophosphate: 2%
Bean pulp: 20%	Mountain flour:1.5%
Imported fish meal: 7%	Composite mineral salt:0.7%
Flour:23%	Decavitamin:0.3%
Yeast:4.5%	Prepowder:1%
