# Supplementary material for: AGGF1 therapy inhibits thoracic aortic aneurysms by enhancing integrin α7-mediated inhibition of TGF-β1 maturation and ERK1/2 signaling
Source: Nat Commun. 2023 Apr 20;14:2265. doi: 10.1038/s41467-023-37809-x (PMC10119315; doi:10.1038/s41467-023-37809-x)
Supplement: Supplementary file 2 — Reporting Summary [file 41467_2023_37809_MOESM2_ESM.pdf]

nature portfolio

Corresponding author(s): Qing K. Wang

Last updated by author(s): 03/13/2023

Reporting Summary

Nature Portfolio wishes to improve the reproducibility of the work that we publish. This form provides structure for consistency and transparency in reporting. For further information on Nature Portfolio policies, see our Editorial Policies and the Editorial Policy Checklist.

Statistics

For all statistical analyses, confirm that the following items are present in the figure legend, table legend, main text, or Methods section.

n/a	Confirmed	
☐	☒	The exact sample size (*n*) for each experimental group/condition, given as a discrete number and unit of measurement
☐	☒	A statement on whether measurements were taken from distinct samples or whether the same sample was measured repeatedly
☐	☒	The statistical test(s) used AND whether they are one- or two-sided<br>*Only common tests should be described solely by name; describe more complex techniques in the Methods section.*
☐	☒	A description of all covariates tested
☐	☒	A description of any assumptions or corrections, such as tests of normality and adjustment for multiple comparisons
☐	☒	A full description of the statistical parameters including central tendency (e.g. means) or other basic estimates (e.g. regression coefficient) AND variation (e.g. standard deviation) or associated estimates of uncertainty (e.g. confidence intervals)
☐	☒	For null hypothesis testing, the test statistic (e.g. *F*, *t*, *r*) with confidence intervals, effect sizes, degrees of freedom and *P* value noted<br>*Give P values as exact values whenever suitable.*
☒	☐	For Bayesian analysis, information on the choice of priors and Markov chain Monte Carlo settings
☒	☐	For hierarchical and complex designs, identification of the appropriate level for tests and full reporting of outcomes
☒	☐	Estimates of effect sizes (e.g. Cohen's *d*, Pearson's *r*), indicating how they were calculated

Our web collection on statistics for biologists contains articles on many of the points above.

Software and code

Policy information about availability of computer code

Data collection	Echocardiography was performed with a Vevo 2100 High Resolution Micro Ultrasound System (Visual Sonics, Canada); Immunohistochemistry and histology of tissues were photographed under a florescence microscope or a confocal microscope; Exposure of immunoblotting was performed in a ChemiDoc system (Bio-Rad, USA). Echocardiography was performed by a researcher who was blinded to the animal's genotypes and treatment status.
Data analysis	Quantification of immunoblotting and immunofluorescence staining were analyzed using Image J (version 1.49).<br>Quantitative real-time PCR analysis was performed using the qPCR SYBR Green Master Mix (Low Rox Plus) (Cat number MQ10201S, Monad, China) on a QuantStudio® 12K Flex Real Time PCR System (Thermo, Waltham, MA, USA).<br>Quantitative data were shown as mean ± SEM. The difference between two groups of variables was compared by the unpaired 2-tailed Student's t-test and comparison of multiple groups (≥3 groups) was performed by one or two-way ANOVA with Tukey's multiple-comparison tests. A P value of <0.05 was considered to be statistically significant.

For manuscripts utilizing custom algorithms or software that are central to the research but not yet described in published literature, software must be made available to editors and reviewers. We strongly encourage code deposition in a community repository (e.g. GitHub). See the Nature Portfolio guidelines for submitting code & software for further information.

Data

Policy information about availability of data

All manuscripts must include a data availability statement. This statement should provide the following information, where applicable:

- Accession codes, unique identifiers, or web links for publicly available datasets
- A description of any restrictions on data availability
- For clinical datasets or third party data, please ensure that the statement adheres to our policy

The data that support the conclusions of this study are provided in the article or the online supplementary data, and also available from the corresponding authors. Source data are provided with this paper.

Human research participants

Policy information about studies involving human research participants and Sex and Gender in Research.

Reporting on sex and gender	The information was provided in Supplementary Table S4.
Population characteristics	The patient characteristics were listed in Supplementary Table S4. TAA patients had a maximum thoracic aortic diameter of between 5.0 to 6.5 cm. The non-TAA study subjects did not have TAA. There was no significant difference for the age, sex, medications and other characteristics between the two groups.
Recruitment	The aortic tissue samples were collected from patients with thoracic aortic aneurysms undergoing surgical resection of the aortic wall and from explanted hearts from cardiac transplantation. The non-TAA thoracic aortic tissue samples were obtained from healthy donors undergoing ascending aortic trimming before heart transplantation. The tissues samples were all from medically-needed procedures, and would normally be discarded after the procedures.
Ethics oversight	This study protocol was approved by the Ethics Committee of Wuhan Union hospital of Tongji Medical College, Huazhong University of Science and Technology (No: [2018]S353).

Note that full information on the approval of the study protocol must also be provided in the manuscript.

Field-specific reporting

Please select the one below that is the best fit for your research. If you are not sure, read the appropriate sections before making your selection.

- [x] Life sciences
- [ ] Behavioural & social sciences
- [ ] Ecological, evolutionary & environmental sciences

For a reference copy of the document with all sections, see nature.com/documents/nr-reporting-summary-flat.pdf

Life sciences study design

All studies must disclose on these points even when the disclosure is negative.

Sample size	Sample size determination was performed before each study using nQuery (n=7 mice per group are needed to observe a significant difference for two groups with means different by two-fold, a power of 80% and p of 0.05).
Data exclusions	No data were excluded from analysis.
Replication	All molecular experiments were performed with at least three independent biological samples. Mouse studies were performed in male and female mice independently, and they provide cross-replication. All attempts at replication were successful.
Randomization	Mice were matched by genotype and age and weight, and then randomly assigned to specific treatment groups. For TAC studies, the mice were randomized into sham groups and TAC groups, and the surgical procedures were then performed for all mice. The survived mice were then treated with AGGF1 or controls.
Blinding	Echocardiography was performed by a researcher who was blinded to the animal's genotypes and treatment status. In other mouse experiments, the researchers were blinded to different groups of mice when collecting samples or data.

Reporting for specific materials, systems and methods

We require information from authors about some types of materials, experimental systems and methods used in many studies. Here, indicate whether each material, system or method listed is relevant to your study. If you are not sure if a list item applies to your research, read the appropriate section before selecting a response.

Materials & experimental systems

n/a	Involved in the study
☐	☒ Antibodies
☐	☒ Eukaryotic cell lines
☒	☐ Palaeontology and archaeology
☐	☒ Animals and other organisms
☒	☐ Clinical data
☒	☐ Dual use research of concern

Methods

n/a	Involved in the study
☒	☐ ChIP-seq
☒	☐ Flow cytometry
☒	☐ MRI-based neuroimaging

Antibodies

Antibodies used	For IHC analyses: anti-AGGF1 (Cat number 11889-1-AP, Lot number 00087864, Proteintech, America), anti-α-SMA (Cat number GB111364, Lot number AC220719046, Servicebio, China), anti-MCP1 (Cat number GB11199, Lot number AC220711037, Servicebio, China), and anti-CD68 (Cat number GB11067,Lot number AC220707009, Servicebio, China).<br>For western blot analyses anti-AGGF1 (Cat number 11889-1-AP, Lot number 00087864, Proteintech, America), anti-β-actin (Cat number AB0035, Lot number F210074, Abway, China), anti-GAPDH (Cat number AB0038, Lot number F076406, Abway, China), anti-p-Smad3 (Cat number CY5140, Lot number F086409, Abway, China), anti-T-Smad3 (Cat number CY5013, Lot number F085504, Abway, China), anti-p-Smad2 (Cat number AP1342, Lot number WH297503, ABclonal, China), anti-T-Smad2 (Cat number CY5090, Lot number F078108, Abway, China), anti-p-ERK1/2 (Cat number CY5277, Lot number F072609, Abway, China), and anti-T-ERK1/2 (Cat number CY5487, Lot number F086408, Abway, China), anti-FLAG (Cat number M185-3L, Lot number 016, MBL, Japan), anti-GFP (Cat number T0005, Lot number #31j8985, Affinity, America), IgG (Cat number A7028, Beyotime, China), anti-LAP-TGF-β1 (Cat number 21898-1-AP, Lot number 00095079, Proteintech, America), anti-integrin α7 (Cat number A14246, Lot number WX660446, ABclonal, China), and anti-mature TGFβ1 (Cat number A2124, Lot number WH292258, ABclonal, China).<br>The secondary antibodies are Goat anti-rabbit (Cat number BL003A, lot number 22181594, Biosharp) or Goat anti-mouse (Cat number BL003A, lot number 22210628 Biosharp).<br>All antibodies were dissolved in 5% (w/v) BSA of the TBST configuration prior to use, in the proportions specified.
Validation	All antibodies were validated for use in IHC/western blot. All validation statements can be found on the respective antibody website:<br>1. anti-AGGF1: https://www.ptgcn.com/products/AGGF1-Antibody-11889-1-AP.htm<br>2.anti-αSMA: https://www.servicebio.cn/goodsdetail?id=3743<br>3.anti-MCP1: https://www.servicebio.cn/goodsdetail?id=1441<br>4. anti-CD68: https://www.servicebio.cn/goodsdetail?id=1350<br>5. anti-β-actin: http://abways.com/showproduct.asp?cid=AB0035<br>6.anti-GAPDH: http://abways.com/showproduct.asp?cid=AB0038<br>7.anti-p-Smad3: http://abways.com/showproduct.asp?cid=CY5140<br>8.anti-T-Smad3 : http://abways.com/showproduct.asp?cid=CY5013<br>9.anti-p-Smad2: https://abclonal.com.cn/catalog/AP1342<br>10. anti-T-Smad2: http://abways.com/showproduct.asp?cid=CY5090<br>11.anti-p-ERK1/2: http://abways.com/showproduct.asp?cid=CY5277<br>12.anti-T-ERK1/2: http://abways.com/showproduct.asp?cid=CY5487<br>13.anti-FLAG: http://www.mbl-chinawide.cn/search012?keyword=M185-3L<br>14.anti-GFP : https://www.affbiotech.cn/goods-6271-T0005-GFP_tag_Antibody.html<br>15.IgG: https://www.beyotime.com/product/A7028.htm<br>16.anti-LAP-TGF-β1: https://www.ptgcn.com/products/TGF-beta-1-Antibody-21898-1-AP.htm<br>17.anti-integrin α7: https://abclonal.com.cn/Datasheet/Antibodies/A14246.pdf?v=1660997815<br>18.anti-mature TGFβ1: https://abclonal.com.cn/catalog/A2124

Eukaryotic cell lines

Policy information about cell lines and Sex and Gender in Research

Cell line source(s)	MOVAS cells, immortalized VSMCs from mouse aorta (ATCC, American Type Culture Collection), and HEK293T cells (kind gift from Dr. Charles Antzelevitch) from ATCC (American Type Culture Collection)
Authentication	All cell lines were authenticated by commercial companies based on morphology, growth curves, and other cell-specific features.
Mycoplasma contamination	Cell lines were routinely tested and found negative for mycoplasma infection.
Commonly misidentified lines<br>(See ICLAC register)	None of the cell lines used in this study is found in the database of commonly misidentified cell lines.

Animals and other research organisms

Policy information about studies involving animals; ARRIVE guidelines recommended for reporting animal research, and Sex and Gender in Research

Laboratory animals	Heterozygous Aggf1+/- knockout (KO) mice and Aggf1 flox mice (Aggf1fl/fl mice) were generated by Cyagen (Suzhou, China). Taglin-Cre mice on a C57B/L6 background were also generated by Cyagen. Male or female Taglin-Cre mice were genetically identified at 8 weeks of age, and were mated with 8-week-old Aggf1fl/fl mice to produce Aggf1smcKO mice.<br><br>Mice were placed in an IVC at a density of 3-5mice per cage in an SPF animal room with a temperature of 22-25℃, a humidity of 50-60%, and a dark/light cycle of 12 hours. Cages were cleaned once a week.
Wild animals	No wild animals were used in this study.
Reporting on sex	Both male and female mice were studied independently.
Field-collected samples	This study did not involve field-collected samples.
Ethics oversight	Animal care and experimental procedures were approved by the Ethics Committee of Huazhong University of Science and Technology. Animal experiments conformed to the guidelines of the Care and Use of Animals for Research by the Ministry of Science and Technology of the P. R. China (2006–398).

Note that full information on the approval of the study protocol must also be provided in the manuscript.
